# A review of catalytic hydrogenation of carbon dioxide: From waste to hydrocarbons

**DOI:** 10.3389/fchem.2022.1037997

**Published:** 2022-10-11

**Authors:** Lingrui Cui, Cao Liu, Benzhen Yao, Peter P. Edwards, Tiancun Xiao, Fahai Cao

**Affiliations:** ^1^ Engineering Research Center of Large Scale Reactor, East China University of Science and Technology, Shanghai, China; ^2^ OXCCU Tech Ltd, Centre for Innovation and Enterprise, Begbroke Science Park, Oxford, United Kingdom; ^3^ Department of Chemistry, Inorganic Chemistry Laboratory, University of Oxford, Oxford, United Kingdom

**Keywords:** catalyst, CO_2_ hydrogenation, light olefin, hydrocarbon fuels, aromatics, Fischer–Tropsch synthesis

## Abstract

With the rapid development of industrial society and humankind’s prosperity, the growing demands of global energy, mainly based on the combustion of hydrocarbon fossil fuels, has become one of the most severe challenges all over the world. It is estimated that fossil fuel consumption continues to grow with an annual increase rate of 1.3%, which has seriously affected the natural environment through the emission of greenhouse gases, most notably carbon dioxide (CO_2_). Given these recognized environmental concerns, it is imperative to develop clean technologies for converting captured CO_2_ to high-valued chemicals, one of which is value-added hydrocarbons. In this article, environmental effects due to CO_2_ emission are discussed and various routes for CO_2_ hydrogenation to hydrocarbons including light olefins, fuel oils (gasoline and jet fuel), and aromatics are comprehensively elaborated. Our emphasis is on catalyst development. In addition, we present an outlook that summarizes the research challenges and opportunities associated with the hydrogenation of CO_2_ to hydrocarbon products.

## 1 Introduction

As one of the most notable and biggest emitted greenhouse gases, CO_2_ has greatly affected the natural environment, leading to global warming (Wang et al., 2011; Sanz-Pérez et al., 2016). Given these recognized environmental concerns, it is imperative to alleviate the CO_2_ crisis in the atmosphere, an ideal solution to which is proposing sustainable routes that convert CO_2_ into valuable products, especially the catalytic pathways (Yao et al., 2020). The process of converting CO_2_ into hydrocarbon products is attracting much more attention in scientific research recently, some of which has proved practicability *via* pilot-scale or industrial applications. Considering the annual industrial production of CO_2_ at around 3,300–3,500 Mt (Andrew, 2018), besides the environmental effects, the CO_2_ conversion process also shows tremendous potential for further industrial amplification and high economic value in industrial application.

However, CO_2_ is a fully oxidized, thermodynamically stable, and chemically inert molecule (Lu et al., 2014). Thus, the key to advancing CO_2_ utilization is to develop a highly efficient and inexpensive catalyst, which will decrease the difficulty of CO_2_ conversion. In recent years, a vast number of researchers have studied the catalysts for the CO_2_ hydrogenation process, and some recent publications have investigated CO_2_ conversion into valuable hydrocarbon products (Porosoff et al., 2016; Yan and Philippot, 2018; Whang et al., 2019; Helal et al., 2020; Ramyashree et al., 2021). Meanwhile, hydrocarbon synthesis *via* hydrogenation of CO_2_ usually favors the formation of short-chain hydrocarbons, such as light olefins (C_2_-C_4_ olefins), rather than long-chain products such as fuel oils. For instance, Zhang et al. (2022) investigated the hydrogenation process from CO_2_ into light olefins *via* Fe-based catalysts *in situ* doped with Mn, which were prepared by the solvent evaporation-induced self-assembly (EISA) method. With the aid of a Ni-supported metallic catalyst, Zhou et al. (2017a) studied the valorization of CO_2_ into methane and the synthesis process of hydrocarbon products, including dimethyl ether, acetic acid, and gasoline, *via* CO_2_ hydrogenation.

### 1.1 Hazards of CO_2_

With the ever-increasing rate of population growth, the daily activities of human beings continue to threaten the earth *via* the excessive emission of greenhouse gases, mainly from the combustion of fossil fuels (Kandaramath Hari et al., 2015; Xu et al., 2017). As the most important greenhouse gas, CO_2_ presents great stability in the atmosphere, which could persist for 100–160,000 years, reflecting that the global warming caused by CO_2_ may last thousands of years (Bong et al., 2017; Prasad et al., 2017; Shukla et al., 2017). Thus, CO_2_ emission is likely to cause great environmental damage such as ocean acidification, which results in other grievous climate changes (Moomaw et al., 2018).

Despite a huge number of challenges brought by CO_2_ to the environment, the amount of CO_2_ emitted by human society has continuously increased year by year. For instance, it is projected that the primary energy demand all around the world will reach about 20 billion tonnes of oil equivalent by 2040, approximately 70% of which is obtained from fossil fuels. Furthermore, more than half of CO_2_ was emitted in the last 50 years due to rapid economic development and urbanization growth, resulting in a record of more than 400 ppm CO_2_ in today’s atmosphere (Ojelade and Zaman, 2021). In addition to the climatic damage, the effects of CO_2_ emissions on the economy are noticeable. Many governments spend a huge amount of money on the energy industry, reducing the CO_2_ emission into the atmosphere (Santos, 2017). Thus, the development of a CO_2_ conversion process could not only relieve the pressure from the greenhouse effect but also generate enormous economic benefits.

### 1.2 Ways of managing CO_2_

In order to limit the rise of temperature to less than 2°C globally (compared to the preindustrial era) by the end of this century according to The Paris Agreement (Santos, 2017), the atmospheric concentration of CO_2_ must be stabilized. Although the easiest way to decrease CO_2_ emissions is to improve the usage of various renewable energies, including solar, geothermal, wind, and hydropower, instead of coal and petroleum, fossil fuels are likely to play a dominant role in the next few decades. Thus, the main two methods proposed to achieve the reduction of CO_2_ concentration are carbon capture and storage (CCS) and carbon capture and utilization (CCU).

During the CCS handling approach, the CO_2_ collected from transportation and industrial activities is transported to a certain area, followed by long-term burial in geological formations, which experienced only a little progress for a long time because of associated problems (Leung et al., 2014). Hence, this method alone could not solve the problem of global CO_2_ emission.

Compared with the CCS process, the CCU process and relevant technologies could convert captured CO_2_ into useful chemicals and fuels, which provides more efficient methods to figure out the global environmental problems (Li et al., 2019a). Considering the inert property of CO_2_ molecules, it becomes very important to prepare intrinsically stable catalysts with high activity and selectivity, which is helpful to provide a distinct understanding of catalytic transformation in the CO_2_ converting process.

### 1.3 Review objective

In the previous sections, the hazards of CO_2_ emissions, both in the environment and economy, are discussed critically, showing that atmospheric concentration of CO_2_ must be controlled by carbon capture and utilization. Therefore, the objective of this review is to discuss the advances in the catalytic hydrogenation of CO_2_ into hydrocarbon products, including short-chain products, fuel oils, and aromatics, especially the development of various catalysts. In addition, the research area that requires further investigation and provides increasing opportunities is discussed finally.

## 2 Various routes for CO_2_ hydrogenation

The traditional CO_2_ conversion process to hydrocarbon products is achieved *via* two indirect ways: 1) the methanol (MeOH) route, in which CO_2_ is initially converted into MeOH followed by the MeOH to hydrocarbon (MTH), or 2) the RWGS-FTS route, in which CO_2_ is converted into hydrocarbons *via* a reverse water gas shift (RWGS) reaction, which converts CO_2_ to CO followed by Fischer–Tropsch synthesis (FTS) to produce long-chain hydrocarbons from CO (Yao et al., 2017). In both two routes, the distributions of various products, including light/heavy olefins, paraffin, and aromatics, can be controlled by catalytic properties and reaction conditions (Cheng et al., 2017; Zhao et al., 2017). In recent studies, the direct route process of CO_2_ hydrogenation, in which CO_2_ is hydrogenated into hydrocarbon products in a one-step reaction, has become the new focus (Zhou et al., 2019a; Ma and Porosoff, 2019; Ronda-Lloret et al., 2019; Ojelade and Zaman, 2021). Compared with traditional routes, the direct route is generally recognized as a more economical and environmentally acceptable process due to its fewer chemical steps, resulting in lower total energy consumed in the entire process (Marlin et al., 2018).

### 2.1 CO_2_ conversion to hydrocarbons *via* the MeOH route

CO_2_ hydrogenation into MeOH, the oxygenate-mediated pathway, has been investigated for decades, combining CO_2_-to-oxygenate (reaction 1) and the subsequent MeOH-to-olefins (MTO) (reaction 2) or MeOH-to-aromatics (MTA) (reaction 3) (Liu et al., 2018a). However, the mechanism is still a hot research topic discussed by a great number of researchers.

$$CTM:CO_{2}\left(g\right)+3H_{2}\left(g\right)\to CH_{3}OH\left(g\right)+H_{2}O\left(g\right),$$ (1)

$$MTO:nCH_{3}OH(g)\to (-CH_{2}-{)}_{n}(g)+nH_{2}O(g),$$ (2)

$$MTA:nCH_{3}OH\left(g\right)\to C_{n}H_{2n}/C_{n}H_{2n+2}\to aromatcis.$$ (3)

For the synthesis of methanol (Wang et al., 2021a), the carboxylate intermediate (*CO_2_) is reacted to formate (*HCOO) species *via* hydrogenation and then is further converted to *H_3_CO. The catalytic system based on Cu/ZnO is one of the most common catalysts used in the CO_2_ conversion to methanol process, providing active sites of CO_2_ hydrogenation on the Cu system. Another commonly used catalyst is In_2_O_3_/ZrO_2_, which could enhance the *HCOO hydrogenation to *H_3_CO, resulting in the promotion of methanol production (Chen et al., 2019a; Jiang et al., 2020a).

The obtained methanol is then converted into olefins *via* dehydration coupling, forming *CH_2_CH and subsequent hydrogenation, ultimately hydrogenating into various chemicals such as paraffin or aromatics. The conversion of MeOH to hydrocarbons is mainly achieved with the aid of a zeolitic catalyst, generally HZSM-5 and/or SAPO-34. With medium acidity, high stability, and great selectivity for light-olefins, SAPO-34 has become the most popular MTO reaction catalyst since 1990 (Liang et al., 1990; Chen et al., 1999). As for the MTA process, ZSM-5 is more applicable due to its strong acidity and large microporous channels, which not only improve the activity of the aromatic synthesis process but also the generation of hydrocarbons with long chains gets enhanced as well (Dang et al., 2018).

Compared with the RWGS-FTS process, which has been investigated more extensively, the products obtained from the MeOH route are not limited by ASF distribution, which is favorable for olefin or aromatic production (Li et al., 2017). Thus, the indirect route has greater potential for further industrial amplification and higher economic value in the industrial application (Centi et al., 2013).

### 2.2 CO_2_ conversion to hydrocarbons *via* the RWGS-FTS route

In the RWGS-FTS route for CO_2_ conversion to hydrocarbons, the reduction from CO_2_ to CO *via* RWGS reaction (reaction 4) followed by a series of Fischer–Tropsch synthesis (reactions 5–6) is required (Dorner et al., 2010).

$$RWGS:CO_{2}\left(g\right)+H_{2}\left(g\right)\to CO\left(g\right)+H_{2}O\left(g\right),$$ (4)

$$FTS:nCO\left(g\right)+2nH_{2}\left(g\right)\to {\left(-CH_{2}-\right)}_{n}\left(g\right)+nH_{2}O\left(g\right),$$ (5)

$${\left(-CH_{2}-\right)}_{n}\left(g\right)\to aromatcis.$$ (6)

In the RWGS-FTS route, CO_2_ is initially absorbed on active phases in the RWGS catalysts (such as Cu and Fe_3_O_4_) and activated to form carboxylate species *CO_2_ (Kattel et al., 2016; Jiang et al., 2020b). Hydrogenated by adsorbed H, the obtained *CO_2_ is converted into *HOCO intermediate, which is then dissociated into *OH and *CO species during the RWGS reaction and hydrogenated into *H_2_O subsequently, desorbing gaseous CO or forming hydrocarbons *via* successive FTS ultimately.

For the reaction route of *CO conversion into hydrocarbons, the *CO species dissociated from *HOCO are initially converted into *HCO, which is subsequently hydrogenated into *CH_x_ species as the precursors for olefins and paraffin by means of a series of processes such as hydrogenation, dissociation, and dehydration. The generated paraffin or olefins could be then converted into aromatics (Tackett et al., 2019).

For the RWGS-FTS reaction route, Fe-based catalysts are most widely used (Herranz et al., 2006; Liao et al., 2007; de Smit et al., 2009; Rønning et al., 2010; Xu et al., 2014), due to their lower activity of methanation under high reaction temperatures, which are favorable for the formation of long-chain hydrocarbons. Among different phases of Fe, Fe_2_O_3_ presents an inferior activity for both FTS and RWGS reactions. Thus, the prepared catalysts need to be reduced in reducing gases to convert Fe_2_O_3_ to active phases of iron (metallic Fe, Fe_3_O_4_, FeO, and iron carbides). The Fe_3_O_4_ is an active component for RWGS reaction, while the metallic Fe and iron carbides could convert CO into hydrocarbons. Furthermore, Fe species need to be doped with appropriate promoters to enhance the catalytic activity and increase the hydrocarbon selectivity. For instance, the alkali metals such as Li, Na, K, Rb, and Cs could be used as electron donors, enhancing CO_2_ adsorption and restraining the H_2_ affinity over a catalytic active site.

### 2.3 Direct hydrogenation of CO_2_

The direct hydrogenation of CO_2_ process is usually described as a chemical process combining the RWGS reaction and the subsequent hydrogenation reaction, in which CO_2_ hydrogenation is achieved *via* a one-step reaction.

$$nCO_{2}(g)+3nH_{2}(g)\to (-CH_{2}-{)}_{n}(g)+2nH_{2}O(g).$$ (7)

Showing the great potential of addressing excessive CO_2_ emission and establishing a carbon-neutrality society, the direct hydrogenation process of CO_2_ to light olefins, liquid fuel hydrocarbons, and aromatics receive greatly sparked enthusiasm from both the academic and industrial worlds, which not only provides a route of preparing high-valued chemicals but also presents feasible cost savings related to the carbon tax, requiring the tandem catalyst containing active sites for the individual reaction step (Gao et al., 2017a; Ramirez et al., 2020; Gambo et al., 2022).

With the aid of a tandem catalyst that has multiple functionalities, the direct conversion of CO_2_ mediates a series of reactions in the RWGS-FTS route (Wang et al., 2021a; Nezam et al., 2021; Saeidi et al., 2021). As mentioned earlier, the Fe-based catalyst, the most widely used catalyst for the RWGS-FTS reaction, is able to mediate the direct hydrogenation process of CO_2_ as well. In addition, zeolite also shows potential to be used in the direct process due to its acid sites, which could regulate the subsequent reaction from intermediates to various hydrocarbons (Dokania et al., 2019; Wang et al., 2020a; Wang et al., 2020b). For the direct hydrogenation process, there are two main challenges faced by the design of catalysts. The first one is the suppression of CO selectivity, which could be hydrogenated into various hydrocarbons *via* FTS reaction, requiring high CO partial pressure produced from RWGS reaction (Li et al., 2017; Tan et al., 2019; Wang et al., 2021b). Another challenge affecting the preparation of hydrocarbon products lies in the formation of CH_4_, showing negative effects on the preparation of high-valued products such as light olefins and liquid fuels, which depends on the H* balance on the surface of the catalyst, while the excessive H* is favorable for the formation of CH_4_ (Weber et al., 2021).

**FIGURE 1:**
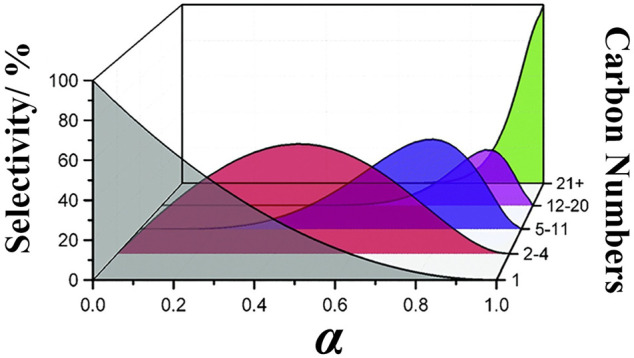
Distribution of FTS process products based on the ASF model.

The distribution of hydrocarbon products in FTS reaction and direct process under equilibrium conditions follows the Anderson–Schulz–Flory (ASF) distribution, in which the selectivity of various hydrocarbon products is limited by their chain growth, as shown in the following equation (Figure 1):

$$M_{n}=\left(1-\alpha \right)\cdot \alpha^{n-1},$$ (8)

where *M*_*n*_ is the molar fraction of *C*_*n*_ hydrocarbon, *n* is the number of carbon atoms in hydrocarbon (n > 1), and *α* is the probability of chain growth (van Der Laan and Beenackers, 1999). The other mathematical expression of the ASF distribution is shown as follows (Prieto, 2017):

$$W_{n}=n{\left(1-\alpha \right)}^{2}\cdot \alpha^{n-1},$$ (9)

$$\ln\left(W_{n}/n\right)=n\ln\alpha +\ln\left[{\left(1-\alpha \right)}^{2}/\alpha \right],$$ (10)

where *W*_*n*_ is the weight fraction of *C*_*n*_ hydrocarbon.

## 3 CO_2_ hydrogenation to light olefins

Among the possible hydrogenation products of CO_2_, light olefins (C_2_^=^-C_4_^=^) can be used for polymer monomer or as an intermediate to synthesize fine chemicals, which is a competitive high-value-added product. Generally, light olefins come from petroleum, which requires a very complicated, high-energy-consuming process, emitting lots of CO_2_ emissions and environmental pollution (Ren et al., 2006; Ren et al., 2008; Centi et al., 2013). The CO_2_ conversion process to light olefins is accompanied by strong competitiveness compared to the traditional process, such as the inexpensive carbon source, simple reaction equipment, and fewer carbon emissions.

In this section, the modification strategies of catalysts that convert CO_2_ into light olefins in recent studies are reviewed. Then, we discuss CO_2_ hydrogenation reaction performance under various reaction conditions. The results of previous research studies are partly shown in Table 1, and details of relevant investigations are discussed in the following parts.

**TABLE 1:** Catalytic performances for the CO_2_ conversion into light olefins.

Catalyst	H_2_/CO_2_ (molar)	Reaction temperature (°C)	Reaction pressure (MPa)	GHSV (gas hourly space velocity)	CO_2_ conversion (%)	Selectivity (%)			Ref
						CO	CH_4_	C_2_^=^-C_4_^=^	
Fe_3_O_4_	3.0	340	1.0	4,800 ml/g·h	27.0	35.9	43.2	5.7	Xu et al. (2019)
Fe/Mn	3.0	340	1.0	4,800 ml/g·h	27.3	30.0	36.0	3.5	
Fe/Na	3.0	340	1.0	4,800 ml/g·h	33.0	20.9	20.7	24.4	
Fe/Na	3.0	320	1.0	4,800 ml/g·h	29.9	24.7	13.2	22.7	
Fe/Na/HZSM-5	3.0	340	1.0	4,800 ml/g·h	30.9	26.4	26.5	3.2	
Fe/Na/HZSM-5	3.0	300	0.2	4,800 ml/g·h	23.8	83.3	56.2	15.3	
Fe/Na/HZSM-5	3.0	340	1.0	4,800 ml/g·h	21.8	40.9	14.7	5.7	
Fe_2_O_3_	3.0	300	1.0	1,140 ml/g·h	18	N/A	37	7.6	Albrecht et al. (2017)
Fe_2_O_3_-CT600	3.0	300	1.0	1,140 ml/g·h	23	N/A	14	46.1	
CuFeO_2_-12	3.0	300	1.0	1,800 ml/g·h	18.1	31.9	3.9	C_2+_:96.1	Choi et al. (2017a)
Cu_2_O-Fe_2_O_3_	3.0	300	1.0	1,800 ml/g·h	15.7	28.9	57.6	C_2+_:42.4	
CuFe_2_O_4_	3.0	300	1.0	1,800 ml/g·h	13.3	28.4	38.3	C_2+_:61.7	
K-Fe15	3.0	270	0.5	2,700 ml/g·h	45	∼13	∼20	∼35	Visconti et al. (2017)
FeCu (0.17)/K (1.0)/Al_2_O_3_	3.0	300	1.1	3,600 ml/g·h	29.3	17	7	C_2+_:76	Wang et al. (2018a)
MIL-53/Fe_2_O_3_	3.0	300	3.0	3,600 ml/g·h	∼21	∼20	∼40	0	Hu et al. (2016)
2 μm ZIF-8/Fe_2_O_3_	3.0	300	3.0	3,600 ml/g·h	∼31	∼18	∼27	∼7	
300 nm ZIF-8/Fe_2_O_3_	3.0	300	3.0	3,600 ml/g·h	∼27	∼21	∼21	∼15	
150 nm ZIF-8/Fe_2_O_3_	3.0	300	3.0	3,600 ml/g·h	∼23	∼23	∼21	∼21	
Fe-Co/K/Al_2_O_3_	N/A	300	1.1	3,600ml/g·s	N/A	∼19	∼25	∼15	Satthawong et al. (2015)
Fe/K/C	3.0	350	3.0	24,000 ml/g·h	38.5	17	∼21	38.8	Ramirez et al. (2018)
Na/Fe	3.0	320	3.0	2,040 ml/g·h	36.8	10.1	7.0	23.4	Liang et al. (2019)
Mn-Na/Fe	3.0	320	3.0	2,040 ml/g·h	38.6	11.7	11.8	30.2	
Fe@NC	3.0	320	3.0	7,200 ml/g·h	28	17.8	26.8	21.2	Liu et al. (2019)
K/Fe@NC	3.0	320	3.0	7,200 ml/g·h	30.6	18.6	16.9	33.1	
Fe_2_O_3_-K	3.0	320	0.5	1,000 ml/g·h	53.9	5.5	26.8	49.9	Zhang et al. (2015a)
Fe-Zn-K	3.0	320	0.5	1,000 ml/g·h	51.0	6	34.9	53.6	
Fe-Co/K-Al_2_O_3_-400	3.0	340	2.0	N/A	49.0	9.4	23.0	37.0	Numpilai et al. (2017)
Fe-Co/K-Al_2_O_3_-500	3.0	340	2.0	N/A	45.9	12.7	21.8	37.1	
Fe-Co/K-Al_2_O_3_-600	3.0	340	2.0	N/A	41.4	14.8	18.5	38.3	
Fe-Co/K-Al_2_O_3_-700	3.0	340	2.0	N/A	37.6	24.5	13.8	33.0	
Fe-Co/K-Al_2_O_3_-800	3.0	340	2.0	N/A	37.2	28.9	13.5	30.2	

### 3.1 Promoters

The addition of alkali metals to the active component of a catalyst is a common method of improving activity and selectivity toward olefins. Doped with alkali promoters such as Na and K as electron donors, the adsorption of CO_2_ on Fe-based catalyst gets improved effectively due to its ability to induce the electronic and structural effects on Fe, enhance the Fe-C bond strength, and inhibit the re-adsorption of olefin, leading to the increment of olefin selectivity and enhancement of C_2_-C_4_^=^ olefin production. Compared with the hydrogenation barrier, the catalysts promoted with alkali metals have lower adsorption energy, making it easier to desorb olefin than hydrogenate undesirably (Satthawong et al., 2015; Xu et al., 2019). For instance, the addition of K and Na to Fe can reduce electrophilicity of Fe efficiently, leading to worse H_2_ affinity of catalyst and a decrease of H concentration on the surface of catalysts, which is favorable for olefin production by hindering the hydrogenation of double bonds in olefins and inhibiting the consequent production of saturated hydrocarbons (Boreriboon et al., 2018a). Another function of alkali metals is to affect the formation of χ-Fe_5_C_2_, which is ascribed to the active phase of the FTS reaction. For example, research studies have shown that K, Cs, and Rb could remarkably accelerate the formation of Fe_5_C_2_ from FeO_x_ and show enhanced olefin production (Ribeiro et al., 2010).

The alkali metal promoters added to the catalyst can affect the activity of catalysts and selectivity of various hydrocarbon products as well. For instance, a study conducted by Satthawong, R and co-workers showed that the mole ratio of olefin/paraffin in products was significantly increased with an increase in the K/Fe atomic ratio in catalysts, affecting the formation of K_2_O species as the electron donor, which is favorable for the reduction of Fe species and the formation of Fe_5_C_2_ (Satthawong et al., 2013). Liu et al. reported that K presented a similar promoting effect on Co-based catalysts prepared, which could change the electron density of active species, weakening the C-O chemical bond and enhancing the Co-C bond, which is beneficial for olefin production (Liu et al., 2021).

Beyond alkali metals, the addition of a transition metal is another effective method for catalyst modification. For instance, Cu could promote the dispersion of Fe particles, leading to the reduction of FeO_x_ and subsequent carburization to generate iron carbide (Zhu et al., 2020). In addition to Cu, CuO is also an effective promoter that improves the activity of catalysts for RWGS because Cu^0^ particles (reduced by H_2_) and FeO_x_ species can be formed during the RWGS reaction, which is beneficial to the formation of active oxygen species on the catalyst surface (Wang et al., 2018a).

The performance of Cu-promoted catalysts also depends on the type of Cu precursor. For instance, the catalyst obtained from delafossite CuFeO_2_, in which Cu is intermediately oxidized to Cu^+^, is beneficial to the formation of the χ-Fe_5_C_2_ phase, leading to the remarkable selectivity for C_5+_ hydrocarbons and high olefin/paraffin ratio in products. Moreover, the catalyst promoted by the spinel CuFe_2_O_4_ precursor only has great selectivity for olefins, in which the Cu in CuFe_2_O_4_ is fully oxidized to Cu^2+^ (Choi et al., 2017a).

The Fe-based catalyst modified by Zn leads to the generation of ZnO and ZnFe_2_O_4_ spinel phases, which leads to an increase in the interaction of Fe-Zn and hinders the sintering of Fe oxides, improving the stability and CO_2_ adsorption of catalysts. Although the interaction of Zn with Fe is unfavorable for C_5_^+^ hydrocarbon production and thereby increases the selectivity for C_2_-C_4_ olefin, excessive Zn promotion (the molar ratio of Zn/Fe is more than 1:1) is unfavorable for olefin production due to the increase in selectivity for CO and CH_4_ promoted by ZnO (Zhang et al., 2015a). With appropriate loading content, the addition of Zn could enhance the activity of catalyst with more active sites on the surface.

Manganese (Mn) and cobalt (Co) are other frequently used promoters in CO_2_ hydrogenation to olefins. As an active phase in the RWGS reaction, MnO_x_ favors the formation of FeO and Fe mixtures which is beneficial for the adsorption of CO_2_. The catalyst-promoted Mn shows greatly improved selectivity toward light olefins due to the availability of strong Mn-Fe surface species, increasing the Fe_5_C_2_ phase content and decreasing the amount of CO adsorption. Hindering the chain-growth reaction, the interaction between Fe and Mn favors the production of olefins, leading to the increase of the olefin/paraffin molar ratio in products. With an increase of loaded Mn from 0 to 5 wt%, the amount of Fe_5_C_2_ in the catalyst increases from 27.1 to 76.8 wt%. However, the amount of Fe_5_C_2_ decreased when the amount of Mn loading was more than 10 wt% (Liang et al., 2019; Jiang et al., 2020b).

### 3.2 Catalyst supports

In addition to promoters, support is also a crucial factor that affects the activity and selectivity of the catalyst due to its ability to interact with active phases during the reaction. Various kinds of support materials have been employed in CO_2_ conversion to light olefins, including metal oxides and metal-organic frameworks (MOFs), which have particular structures beneficial for heat and mass transfer during the reaction (Ronda-Lloret et al., 2019). With mesoporous or macroporous structures, these supports can form active species over catalyst surface, which is able to induce the electronic properties of catalysts, improving their catalytic performance.

With the ability to affect the crystallite size of catalysts, improve the reducibility of Fe_2_O_3_ phases, and control the selectivity for various products, Al_2_O_3_ is one of the most widely used metal oxide supports for Fe-based catalysts (Numpilai et al., 2019). For instance, Al_2_O_3_ with a large pore size could promote the Fe_2_O_3_ reduction to metallic Fe, enhancing CO hydrogenation. With high surface basicity, which could improve CO_2_ adsorption during the reaction, ZrO_2_ is considered one of the most active supports among various metal-oxide supports. This is also suitable for CeO_2_ support which could affect the ratio of olefin/paraffin in products (Xu et al., 2019). With great redox properties, CeO_2_ support is capable of modifying the properties of catalysts and promoting the reduction of FeO_x_ species into active phases, which could be enhanced by using catalysts with nano-cube and nanoparticle structures (Liu et al., 2017).

In particular, metal-organic framework (MOF) is a promising catalyst support due to its large CO_2_ adsorption capacity, high surface areas, and hydrothermal tolerance (Liu et al., 2017), which has also been investigated for CO_2_ to light olefin (CTLO) process. For example, the Fe-based catalyst coated ZIF-8 presents a remarkable selectivity for light olefins, whereas the catalysts supported MIL-53 and g-Al_2_O_3_ with high acidity are unfavorable for olefin production, leading to the enhancement of CO_2_ hydrogenation into alkanes (Hu et al., 2016). Additionally, the selectivity for olefin decreases with the size of supports and the amount of coated graphitic carbon, which might affect the diffusion of reactants, intermediates, and products, consequently changing the distribution of various products.

In addition the supports shown earlier, TiO_2_ and SiO_2_ also have potential as catalyst supports in the CO_2_ hydrogenation process. With oxygen vacancies on the surface, TiO_2_ provides additional sites for CO_2_ adsorption, favorable for the formation of bridged carbonate species, which consequently decompose into carbon intermediates for the C-C coupling reaction (Tumuluri et al., 2017; Boreriboon et al., 2018b). In contrast to other support materials, SiO_2_ could facilitate the dispersion of Fe species, resulting in the suppression of aggregation of active iron particles, which decreases the conversion of CO_2_, which is favorable for methane formation (Samanta et al., 2017).

### 3.3 Preparation methods

In addition to using different supports and promoters shown earlier, the preparation methods are another important factor that influences the CO_2_ conversion reaction performance. Albrecht, M. et al. utilized a cellulose-templated synthesis method to prepare a non-doped Fe_2_O_3_ catalyst, which shows a greater catalytic performance of CO_2_ conversion and higher olefin selectivity than Fe_2_O_3_ prepared by precipitation. The superior performance of non-doped Fe_2_O_3_ may be due to its high content of Fe carbides (around 80%), consisting of χ-Fe_5_C_2_, ε-Fe_2.2_C, and/or θ-Fe_3_C, much higher than that of precipitated Fe_2_O_3_ (about 30% χ-Fe_5_C_2_), favoring chain growth and suppressing the formation of CH_4_ (Albrecht et al., 2017).

The structure and oxidation state of Fe are greatly affected by the duration of the calcination. For instance, Fe_3_O_4_/γ-Fe_2_O_3_ phases are generated under fast calcinations, inducing the formation of Fe carbide phases with high activity (Visconti et al., 2017). In addition to calcination time, temperature is another effective factor. For Fe-Co/KAl_2_O_3_, the increase in calcination temperature could enhance the interaction of Fe oxide with other metal oxides from supports and promoters. On the other hand, the increment of calcination temperature leads to the suppression of Fe oxide reducibility, decreasing the activity of Fe carbides (Numpilai et al., 2017). Additionally, engineered nanostructures could also be used to direct the reactions of the CO_2_ hydrogenation process.

For instance, Liu and co-workers prepared Fe-based catalysts overcoated with ZnO and nitrogen-doped carbon (NC), which exhibited enhanced catalytic activity, stability, and high selectivity toward light olefins and C_5_^+^ hydrocarbons (Liu et al., 2019).

### 3.4 Optimization of reaction conditions

The reaction conditions, including temperature, pressure, velocity speed, and composition of the feed gas, play an important role in the CO_2_ conversion process by affecting the reaction activity and distribution of various products. For instance, as a decreased mole reaction, the high reaction pressure is favorable for CO_2_ hydrogenation thermodynamically. In addition, high reaction pressure favors the generation of *C species from CO dissociation on the catalyst surface, facilitating the formation of Fe carbide phases (Xu et al., 2019). Thus, the high reaction pressure is considerable under the premise of catalyst stability.

CO_2_/H_2_ ratio in feed gas also affects the CO_2_ hydrogenation process, which could control the distribution of various products, especially the ratio of olefins to paraffin. For instance, CO_2_ conversion presents higher activity with a lower CO_2_/H_2_ ratio. Additionally, the secondary hydrogenation of olefins gets suppressed under high CO_2_ pressure, resulting in the improvement of light olefin selectivity (Ramirez et al., 2018; Jiang et al., 2020b). Another important parameter is the reaction temperature. In general, the increment of the reaction temperature is favorable for both the activity of RWGS and FTS reactions. In addition, high reaction temperature has positive effects on olefin selectivity because it is favorable for chain growth in FTS, leading to the increase of selectivity toward light olefins.

## 4 CO_2_ hydrogenation to gasoline

The CO_2_ conversion process to selective hydrocarbons is essential for renewable energy demands, contributing to the alleviation of excessive CO_2_ emissions. Amongst all kinds of gaseous and liquid hydrocarbons from C_1_ and C_2_ up to > C_21_, the production of hydrocarbon fuels has attracted worldwide attention from society due to its ability to produce truly clean energy (Yao et al., 2020). In this section, various modifications of CO_2_ hydrogenation catalysts that produce gasoline range hydrocarbons (C_5_-C_11_), both zeolite-based and non-zeolite catalysts (including photocatalyst), are reviewed.

### 4.1 Zeolite-based catalysts for CO_2_ conversion into gasoline

**FIGURE 2:**
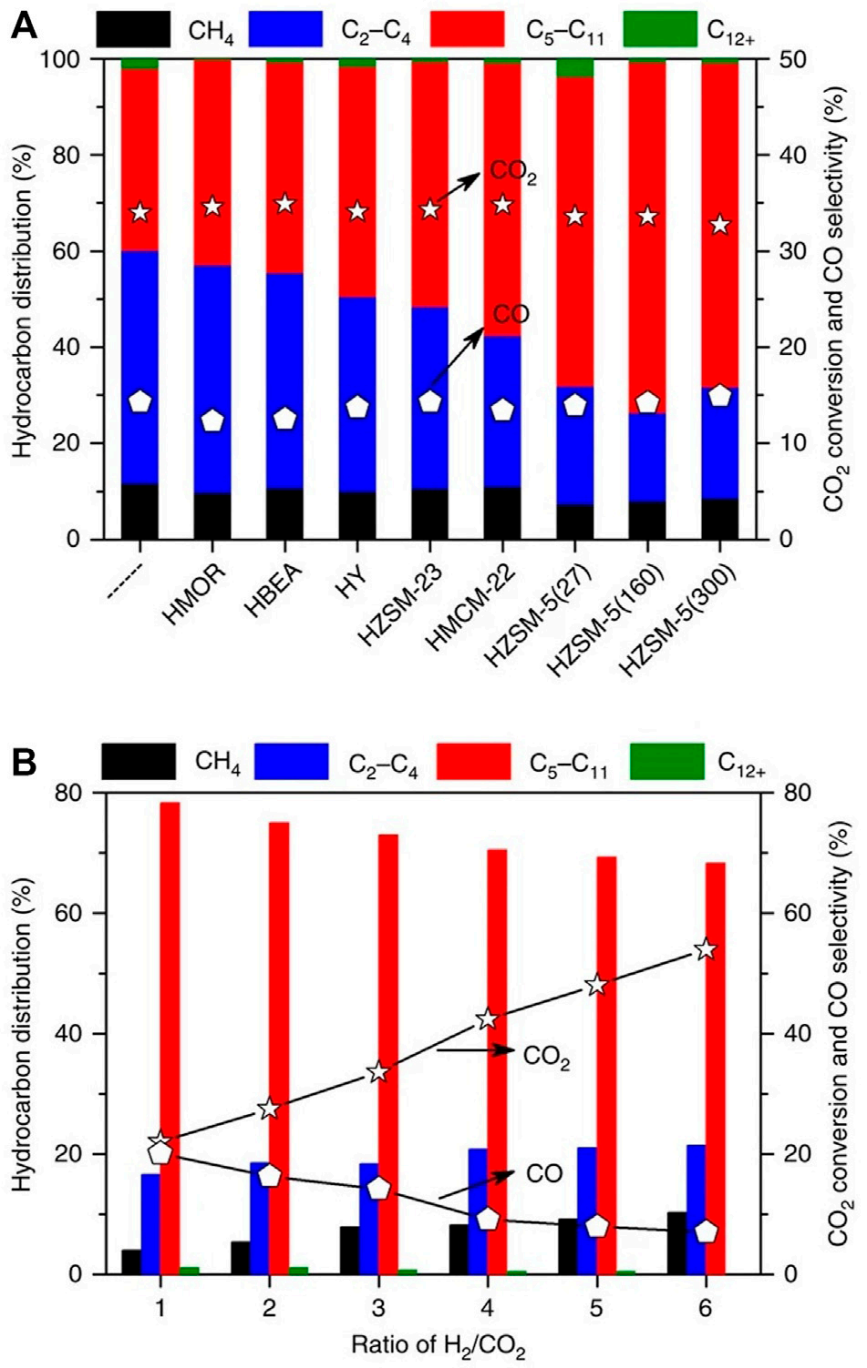
CO_2_ conversion and different product selectivity of various catalysts (Wei et al., 2017). **(A)** Na-Fe_3_O_4_/zeolite catalysts. **(B)** Different H_2_/CO_2_ ratios over Na-Fe3O4/HZSM-5 catalyst.

With variable pore structures that could improve the catalytic performance, zeolite-based catalysts have continued attracting a lot of interest in the CO_2_ hydrogenation process into gasoline (Ma et al., 2016; Zhou et al., 2017b; Poursaeidesfahani et al., 2017; Arslan et al., 2019). For instance, Ni et al. prepared a catalyst system with a combination of ZnAlO_x_ and HZSM-5 (Ni et al., 2018). With low CO_2_ conversion (∼10%), the obtained catalyst presented high selectivity for CO of the total product (57.4%), with HC fractions that are mainly aromatics (∼74%). Gao and co-workers used In_2_O_3_ instead of Zn-Al oxide to prepare the HZSM-5 catalyst, aiming to improve gasoline fraction selectivity (Gao et al., 2017b). Although presenting high selectivity for overall HC production (∼78%), the obtained catalyst also had a high selectivity for CO (∼45%), which is likely to limit the overall selectivity of the goal product. Similarly, the catalyst system combining HSAPO-34 and In_2_O_3_-ZrO_2_ was reported by the same group (Gao et al., 2017a). With the aid of light olefin (C_2_-C_5_) production, the prepared catalyst had an exceedingly high selectivity of CO production (>80%), greatly limiting the distribution of the products and application of the tested catalyst. Fujiwara and co-workers prepared a series of multi-metal CO_2_ hydrogenation catalysts, such as HY zeolite loaded with Cu-Zn-Cr, testing the distribution of various products including olefins, paraffin, and aromatic components (Fujiwara et al., 1995). With the high conversion of CO_2_ (33.5%), the results showed that hydrocarbon selectivity was poor, whereas CO reached a selectivity of 80%. Wang and co-workers investigated the effects of zeolitic topology, such as H-BEA and ZSM-5 on the activity of Fe-Zn-Zr catalysts (Wang et al., 2016). The results showed that H-BEA supporting co-structured catalyst had better activity (and >68% selectivity) than that supported on H-ZSM-5 and HY, and the best selectivity of hydrocarbon products was in the production of isoalkanes (∼81%). Using Na modified Fe_3_O_4_ supported on ten-membered ring (MR) channels, Wei et al. prepared gasoline fraction (C_5_-C_11_) from CO_2_ hydrogenation with a best selectivity of 61% and a CO selectivity of only 15%, showing that olefin was oligomerized into gasoline and diesel range hydrocarbons (C_5_-C_11_) (Wei et al., 2017) (Figure 2).

Rongxian et al. tested the catalytic performance of Fe/Zn catalyst loaded on different zeolites modified by various metals, including La, Al, Mn, Cr, and Zr, the results of which showed that the activity of CO_2_ hydrogenation catalysts was greatly affected by the bulk properties of modifiers (Rongxian et al., 2004). For instance, the catalyst modified by La presented the worst gasoline iso-alkane yield (less than 26%) whereas the Zr-modified catalyst showed the highest iso-alkane selectivity (about 55%). Similarly, Tan and co-workers used Cr, Al, Ga, and Zr to modify the HY zeolite-loaded bi-metallic catalyst, converting CO_2_ into i-C_4_H_12_. Amongst different kinds of prepared catalysts, the Fe-Zn-Zr catalyst loaded on the HY presented the highest yield for i-C_4_H_12_.

Wang and co-workers investigated the distribution of gasoline range hydrocarbon products, including alkane, alkene, and aromatics, using different zeolite-based catalysts (Wang et al., 2020a). As per the experiment results, the alkenes had the highest selectivity by using MOF-synthesized Na-Fe/C catalysts. Multifunctional Na-Fe-C and Na-Fe/C catalyst systems coupled with acidic H-ZSM-5 zeolite presented the best aromatics yield (30.9%), where the CO_2_ was directly converted into aromatics. Additionally, the molar ratio of alkane to alkene in products dramatically decreased from 5.63 to 0.68 by adding H-ZSM-5, indicating that acidic zeolite led to the conversion of alkene to aromatics *via* dehydrogenation and cyclization reaction. For instance, the molar ratio of isoparaffin to paraffin in the products of Na-Fe-C catalyst loaded on H-ZSM-5 was 3.56, verifying the precise adjustment of the effects of acidic zeolites on the product distribution. Furthermore, catalysts loaded on alkaline-treated H-ZSM-5 presented an increment in aromatics selectivity and a decline in the selectivity for C_5+_ non-aromatics.

**FIGURE 3:**
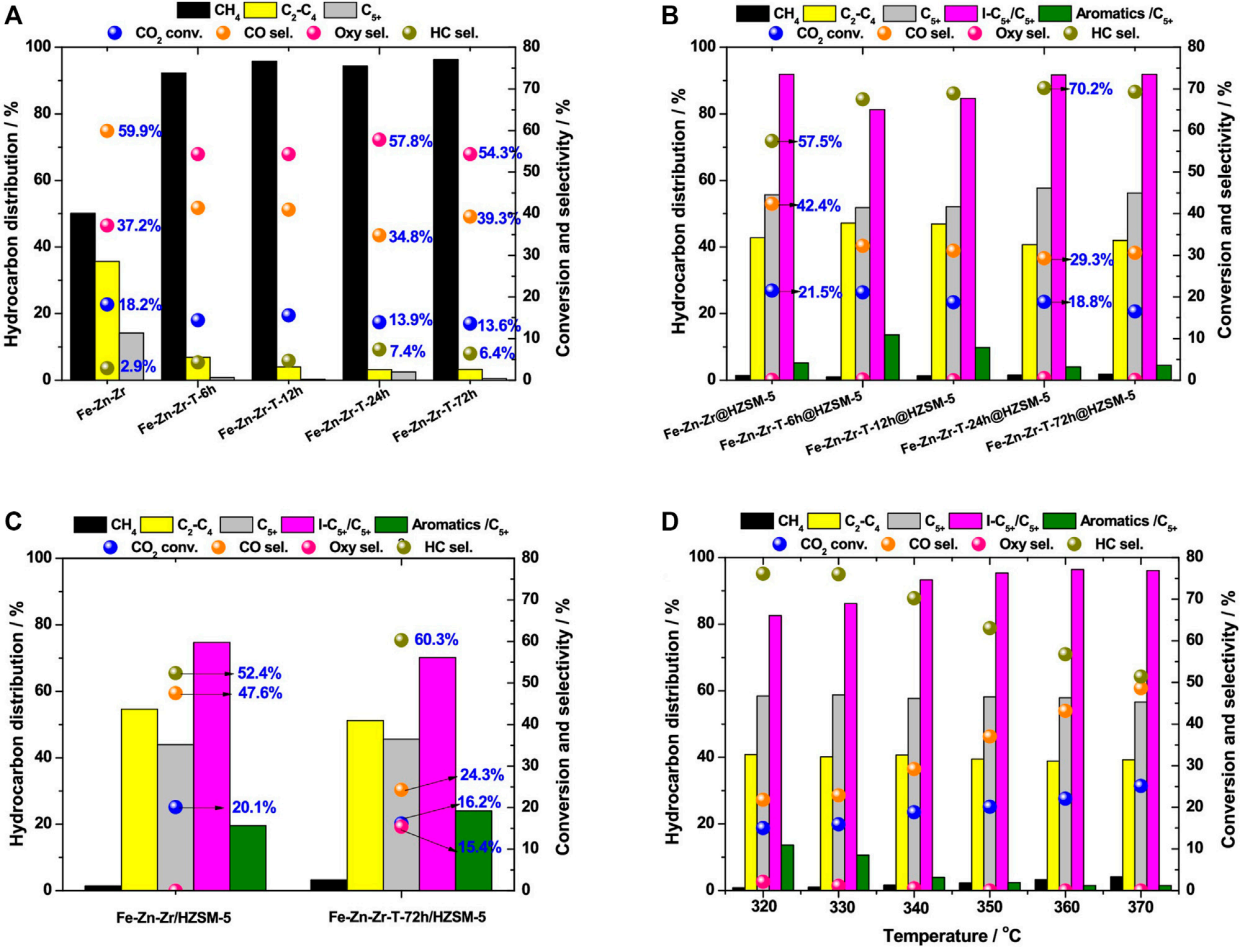
CO_2_ conversion and selectivity for various products over different catalysts (Wang et al., 2021b). **(A)** Fe-Zn-Zr and Fe-Zn-Zr-T-x oxides. **(B)** Fe-Zn-Zr@HZSM-5 and Fe-Zn-Zr-T-x@HZSM-5 core shell. **(C)** Fe-Zn-Zr/HZSM-5 and Fe-Zn-Zr-T-72 h/HZSM-5 granular mixing. **(D)** Fe-Zn-Zr-T-24 h@HZSM-5 core shell under different reaction temperatures.

Wang et al. (2021b) prepared a novel catalyst *via* the combination of TPABr solution-treated metal oxide (Fe-Zn-Zr-T) and HZSM-5, which is used in the CO_2_ hydrogenation process to produce liquid hydrocarbon fuel with high quality. With the aid of hydrothermal pretreatment, both Zn from metal oxide and Br from TPABr solution get enriched in the catalyst, leading to an increment in the number of oxygen vacancies. Thus, the ratio of H_2_ to CO_2_ adsorption gets remarkably increased due to the surface properties of the catalyst, resulting in the enhancement of the adsorption rate of HCOO* species and desorption strength of CH_3_O* species, favorable for the formation of long-chain hydrocarbons (Graf et al., 2009; Kattel et al., 2017; Li et al., 2019b). With the decrease in the molar ratio of Fe to Zn-Zr, the content of HCOO* and CH_3_O* species increased dramatically, leading to a much higher selectivity for C_5_^+^ hydrocarbons on the obtained catalyst (Fe-Zn-Zr-T@HZSM-5). With a CO_2_ conversion of 18%, the isoalkane in C_5_^+^ hydrocarbons reach a maximum selectivity of 93% (Figure 3).

Guo and co-workers prepared a series of Fe/Co catalysts loaded on Y-zeolites containing different metals (such as Ce, K, and La) to convert CO_2_ into linear-alpha olefins (LAOs) (Guo et al., 2019). The research results showed that the content of activated carbide (Fe_5_C_2_) in catalysts was greatly affected by ion exchange strategies, resulting in the differences in activity. With the highest selectivity of C_4+_ alkenes (45.9%), the method of hetero-atom doping presented the best reaction performance with a CO_2_ conversion of 25.9%. Furthermore, the Fe/Co catalyst loaded on K^+^ exchanged zeolite showed better LAO selectivity than other catalysts, with a maximum conversion of 78.9%.

In order to convert CO_2_ into light hydrocarbon fuels with ideal selectivity at mid-low temperature, Ramirez et al. prepared a catalyst by combining ZrS catalyst loaded on the SAPO-34 and the Fe catalyst (such as Fe_2_O_3_@KO_2_), which presented a temperature gap with CO_2_ conversion (Ramirez et al., 2020). The research results indicated that the selectivity of light olefins was significantly affected by the properties of zeolites. In addition, the ZrS layer was favorable for the cracking of heavy hydrocarbons (C_5+_) generated on the Fe phases, covering the shortage of conventional zeolites. With a CO_2_ conversion of up to 50%, the yield of light olefin reached 20%. Under the reaction conditions of 375°C, 30 bar, and H_2_/CO_2_ = 3 and 500 ml/g·h with a light olefin space-time yield of 10.4 mmol/gcat·h, the total selectivity of C_2_-C_4_ olefin reached 40–45%, with a CO selectivity of only ∼16%. The investigation results also showed a slight increment of C_2_-C_4_ light olefin selectivity from 41% to 43% by the combination of Fe_2_O_3_@KO_2_ catalyst with SAPO-34, compared with a common catalyst system.

### 4.2 Non-zeolite catalysts for CO_2_ conversion into gasoline

For non-zeolite catalysts, Fe-based catalysts are one of the most common catalysts for CO_2_ conversion into hydrocarbons. Amoyal and co-workers synthesized a series of K-modified Fe catalysts, such as Fe-Al-O and Fe_5_C_2_, to synthesize gasoline hydrocarbons range fuel (C_5+_) and investigated the catalytic performance of various catalysts (Amoyal et al., 2017). The research results indicated that the addition of K incorporated on Fe-Al-O spinel generated oxygen vacancies on catalysts, leading to the enhancement of the reducibility of catalyst particles. Generally, high content of K loading would decrease the activity of catalysts. However, the K loading on Fe_5_C_2_ was favorable for the improvement of CO_2_ hydrogenation activity to hydrocarbons with a yield of ∼100%. A similar result was obtained from the results reported by Liu and co-workers, in which a Fe-based catalyst was prepared by using a metal-organic framework (MOF) (Zhou et al., 2017b). Compared with the Fe catalyst loaded on Al_2_O_3_ which had a CO_2_ conversion of less than 26% and a bad stability for less than 7 h, the novel catalyst showed better activity (∼40% CO_2_ conversion) and improved stability (>30 h). The results further indicated the negative effects of Fe content on catalytic activity. With the increase of Fe amount from 10% to 30%, the CO_2_ conversion decreased from 26% to less than 20%. Kangvansura and co-workers investigated the effects of nano-Fe/N-doped CNT on the catalytic performance of CO_2_ hydrogenation catalysts (Kangvansura et al., 2017). Although a nano-Fe/N-doped CNT catalyst without modification reached a CO_2_ conversion of 38%, the Fe sintering in catalysts led to poor stability, limiting its application. With the addition of nano-Fe/N-doped CNT and other metal modifiers such as K and Mn, the stability of the CO_2_ conversion process was greatly improved, during which the light olefin was shifted to C_5+_ hydrocarbons. In a similar study, Wang et al. prepared Fe-Zn catalysts modified by K *via* different methods to synthesize C_5+_ hydrocarbons (Wang et al., 2018b). The results showed that catalysts prepared by the hydrothermal route presented higher selectivity for C_5+_ (>30%), while the co-precipitation route is favorable for the improvement of both CO_2_ conversion and hydrocarbon selectivity, with lower C_5+_ selectivity.

Kim et al. prepared a series of CO_2_ hydrogenation catalysts by adding mesoporous bimetallic spinel oxide (MAl_2_O_4_, where M = Mg, Co, Cu, and Zn) and investigated the catalytic performance of the obtained catalysts under high H_2_ pressure at a reaction temperature range from 300 to 400°C (Kim et al., 2020). Despite the inferior selectivity for most catalysts that produced CO as major products (>78%), CoAl_2_O_4_ presented a much better performance that produced CH_4_ as the major product with a selectivity of 80%. The research result indicated that the activity of spinel oxide catalyst depends on its type. The reaction temperature was another significant factor affecting the catalytic performance of spinel oxide catalysts. For instance, CuAl_2_O_4_ showed higher CO_2_ conversion (25.8%) at 300°C than other catalysts under the same temperature: CoAl_2_O_4_ (16.5%) >ZnAl_2_O_4_ (7.0%) >MgAl_2_O_4_ (5.1%), indicating that Co had the best hydrogenation activity, while Mg was the weakest active component.

**FIGURE 4:**
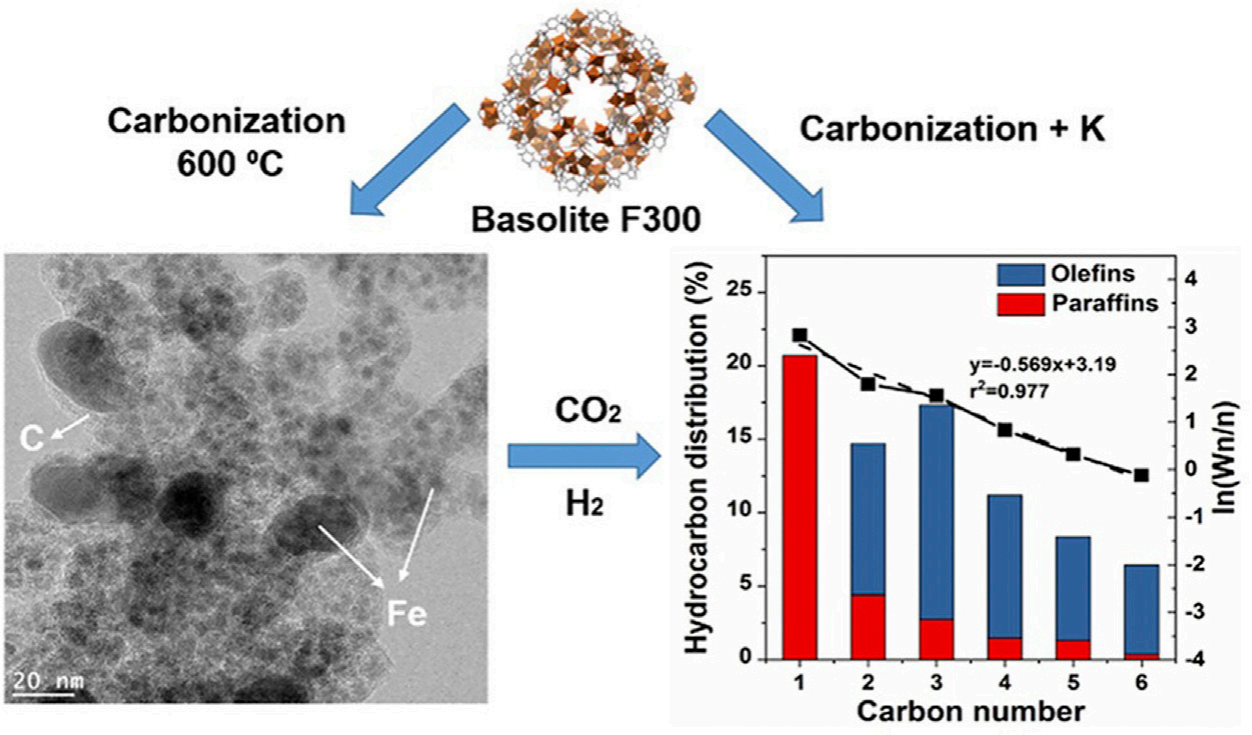
Morphological characterization and product distributions of the Fe MOF (metal-organic framework)-mediated catalyst (Ramirez et al., 2018).

As highly porous crystalline materials, the MOFs and COFs have been utilized for the preparation of CO_2_ conversion to hydrocarbons in recent studies. Despite inferior total CO_2_ conversion, catalysts over MOFs presented great selectivity of certain hydrocarbons. For instance, Tarasvo and co-workers synthesized Co/Al nanohybrid catalyst loaded on microporous MOF Al by an MW-assisted method (Tao et al., 2016). The obtained catalyst showed bifunctionally improved activity of CO_2_ hydrogenation to CO and hydrocarbon fuel production from CO. Under the reaction conditions of 340°C, 30 atm, 800 h^−1^, and H_2_/CO_2_ = 2.7, the optimized reaction reached a CO_2_ conversion of 37.5% and a product distribution of 53.2% CH_4_, 24% C_2_-C_4_ hydrocarbons, and 22.7% C_5+_ hydrocarbons. Adding MOF as a precursor, Ramirez and co-workers prepared a series of Fe-based CO_2_ hydrogenation catalysts modified by various elements (Ramirez et al., 2018) (Figure 4). The activity and selectivity of novel catalysts were investigated for short-chain olefin production. Compared with catalysts modified by other metals, the K-modified catalyst presented a better activity of CO_2_ hydrogenation and higher selectivity for C_2_-C_6_ olefins, increasing from 24% to 0.7%–35% and 36%, respectively. The optimized reaction conditions were 320°C, 30 bar, 2,400 ml/g·h, and H_2_/CO_2_ = 3. With the aid of extensive characterization, the effects of K on the improvement of the catalytic performance of modified catalysts were investigated. Initially, the addition of K could balance the amount of different Fe phases (such as Fe oxide and Fe carbide) that are favorable for CO_2_ hydrogenation. Additionally, K could effectively enhance the absorption of CO_2_ and CO and reduce the affinity of H_2_, leading to the improvement of olefin selectivity. Based on the TEM analysis results, the Fe particles in catalysts are confined within the highly porous carbon matrix with an average size of 4.4 nm. Furthermore, the carbonization led to the reduction of material porosity from 924 to 243 m^2^/g. The hydrocarbon product distribution showed that the selectivity of C_2_-C_6_ olefins significantly improved after K modification, while other promoters were unfavorable for olefin production. Under a reaction temperature of 350°C, optimized CO_2_ conversion and C_2_-C_6_ olefin selectivity were obtained with 38.5% and 38.8%, respectively.

Qadri et al. prepared Ru-Fe nanoparticle (NP) catalysts *via* ionic liquids (ILs) to achieve selective hydrogenation of CO_2_ to fuel range heavy hydrocarbons under normal reaction conditions (Qadir et al., 2018). The results indicated that the NPs presented the excellent activity to produce long-chain hydrocarbons, reaching a CO_2_ conversion and a C_7_-C_21_ selectivity of 12% and 10%, respectively. During the reaction, the diffusion and residence time of both intermediates and products were controlled by the cage around the Ru-Fe NPs formed by ILs. In addition, the CO_2_ conversion rate was not affected by the rise in reaction temperature, which, however, would lead to the change of heavy hydrocarbon selectivity. Another study investigated by Qadri reported the CO_2_ hydrogenation process into light hydrocarbons by using bimetallic Ru/Ni nanoparticles, composed of Ru-rich shells and 2–3 nm Ni-rich cores, under optimized reaction conditions of 150°C, 8.5 bar, and H_2_/CO_2_ = 1:4, with a ratio of Ru/Ni nanoparticles of 3:2 in hydrophobic ILs (Qadir et al., 2019). By using an obtained catalyst, 30% of CO_2_ was converted into hydrocarbons with a distribution of 79% alkanes, 16% olefins, and 5% CH_4_.

As semiconductor materials, photocatalysts are activated in the presence of light irradiation and described as light-photon catalysis to cause catalytic reactions, which have been used in different methods such as chemical oxidation and air purification (Khan et al., 2017). Amongst different kinds of semiconductor photocatalysts, metal oxides such as TiO_2_ and ZnO have gained considerable attraction due to their low cost and high reduction potential, which could be used as catalyst materials for CO_2_ hydrogenation (Ramyashree et al., 2021). In recent studies, electroreduction of CO_2_ has been investigated to prove the mechanism of propanol formation (Veenstra et al., 2020), which also implies the formation parameters during the CO_2_ reduction process to higher hydrocarbons, which is favorable for the design of catalysts to improve selective product selectivity. The mechanism of photocatalytic CO_2_ conversion involves multielectron transfer and the corresponding redox potentials for various products, as shown in reactions from 11 to 15 (Low et al., 2017; Fu et al., 2018).

$$CO_{2}+2H^{+}+2e^{-}\to HCOOH\left(E_{redox}=-0.20V\right),$$ (11)

$$CO_{2}+2H^{+}+2e^{-}\to CO+H_{2}O\left(E_{redox}=-0.12V\right),$$ (12)

$$CO_{2}+4H^{+}+4e^{-}\to HCHO+H_{2}O\left(E_{redox}=-0.07V\right),$$ (13)

$$CO_{2}+6H^{+}+6e^{-}\to CH_{3}OH+H_{2}O\left(E_{redox}=+0.03V\right),$$ (14)

$$CO_{2}+8H^{+}+8e^{-}\to CH_{4}+2H_{2}O\left(E_{redox}=+0.17V\right).$$ (15)

As an inert molecule, CO_2_ is enormously hard to convert into chemicals at room temperature, making it difficult to achieve the photoreduction of CO_2_. In addition to the reaction temperature, the photocatalytic process of CO_2_ conversion is also influenced by other factors, including charge carrier’s separation, band gap energy matching, and photocatalyst basicity (Liu et al., 2012; Mori et al., 2012; Li et al., 2014).

With suitable CB electron, the titania shows great potential to be used for photocatalytic conversion of CO_2_ to hydrocarbons due to its excellent adsorption of visible light and greatly enhanced activity of CO_2_ photoreduction to hydrocarbons (Zhang et al., 2011; Li et al., 2012; Gao et al., 2020a). A lot of efforts have been made to achieve the enhancement of TiO_2_ photocatalysis. For instance, a variety of studies have reported the photocatalytic performance of TiO_2_ doped with non-metals (Asahi et al., 2001; Li et al., 2005; Yu et al., 2005; Schneider et al., 2014) or transient metal ions (Ola and Mercedes Maroto-Valer, 2014; Ola and Maroto-Valer, 2016), significantly extending the light adsorption range of TiO_2_ and enhancing the activity of CO_2_ photoreduction. Additionally, TiO_2_ co-doped with metal and non-metal presented the ability to improve the photoreduction of CO_2_ under visible light. Fan and co-workers prepared TiO_2_ doped with Ni^2+^ and N and investigated the synergistic effect, achieving a MeOH yield of 3.59 μmol/g·h at visible light (400–780 nm) (Fan et al., 2011). Furthermore, a variety of recent studies have prepared hydrocarbon fuels with the aid of a hybrid photocatalyst, overcoming the effects of by-products ranging from CO to CH_4_. For instance, Cronin and co-workers used a TiO_2_ photocatalyst decorated with plasmonic Au NPs for CO_2_ reduction under UV (Hung et al., 2015). The product prepared from the hydrogenation process changes with the frequency of ultraviolet radiation. As the only product, methane reached a yield of 22.4 μmol/g·h under visible light (532 nm). When the UV range changed to 254 nm, a series of products including C_2_H_6_, CH_3_OH, and HCHO was obtained. In addition to Au, Ag is another material that could be pelletized to doping on TiO_2_ photocatalyst. Liu and co-workers used TiO_2_/Ag-NP nanowire films to prepare MeOH (Liu et al., 2015a). With 2.5 wt% Ag/TiO_2_, MeOH production reached 22.4 μmol/g·h under UV-Vis light radiation, 9.4 times higher than that using pure TiO_2_ (Liu et al., 2014). In addition, other metal NPs (including Cu, Ru, Ga, and Cd) have also been used for the process of CO_2_ photocatalytic reduction (Marcì et al., 2014; Liu et al., 2015b; Jun et al., 2019).

In addition to experimental studies, simulation is another significant method to investigate the CO_2_ conversion process into various hydrocarbons. Based on the results of density functional theory (DFT) studies, Peterson and co-workers demonstrated that the formation of CHO* intermediate *via* the protonation of adsorbed CO* was the controlling step of the electrochemical CO_2_ reduction process (Peterson et al., 2010). The study results reported by Lim and co-workers indicated that structural and electronic properties of Cu could be modified by defective graphene-supported Cu-NP, achieving the enhancement of CO_2_ electrochemical reduction to hydrocarbon fuels (Lim et al., 2014).

Subramanian et al. synthesized a multi-metallic greatly disordered nano-crystalline alloy (AuAgPtPdCu) to convert CO_2_ into gaseous hydrocarbon fuels, associated with the activity of highly disordered alloy simulated by DFT (Nellaiappan et al., 2020). Azofra et al. used DFT to simulate the activity of nitride meshes doped with beryllium (Azofra et al., 2016). The results indicated that the obtained highly reactive material produced π-hole, leading to a decrease in CO_2_ hydrogenation activity, which is favorable for the improvement of CH_4_ selectivity.

## 5 CO_2_ hydrogenation to jet fuels

In addition to gasoline, jet fuel is another widely used liquid fuel in the modern transportation system. As the aviation fuels used in gas turbine-powered aircraft, the main components of jet fuel are linear and branched alkanes and cycloalkanes with a carbon number from C_8_ to C_18_, the ideal carbon chain length of which is C_8_ to C_16_ (Kallio et al., 2014). Like other hydrocarbon fuels, the production of jet fuel from renewable energy not only effectively alleviates environmental problems caused by tremendous CO_2_ emissions but also the excessive dependence of human society on fossil fuels is reduced as well (Gao et al., 2020b). In this section, the results of recent academic studies converting CO_2_ into jet fuel are reviewed, focusing on the modification and preparation methods of catalysts.

### 5.1 Promoters

With high RWGS activity, the Fe-based catalyst is a common FTS catalyst used for long-chain hydrocarbon production (Owen et al., 2016; Shi et al., 2018). For instance, Albrecht et al. (2017) used Fe_2_O_3_ catalyst synthesized by the cellulose templated method for the CO_2_ hydrogenation process, achieving a CO_2_ conversion of 40% under the optimized operation conditions of 1.5 MPa and 350°C, with a selectivity for CH_4_, C_2_-C_4_, and C_5_^+^ hydrocarbons of 12%, 36%, and 36%, respectively. Aiming at improving the selectivity for long-chain hydrocarbons, the introduction of promoters, such as alkali metals, to the active component of catalyst formulation is a common modification method. Zhang et al. (2021) prepared a novel CoFe alloy catalyst modified by Na to achieve direct CO_2_ hydrogenation to jet fuel range hydrocarbons. Compared with the original CoFe catalyst without a promoter, which consumes 19.6% CO_2_ with a high CH_4_ selectivity of 70.3%, the Na-modified catalyst had a significantly decreased CH_4_ selectivity and a remarkably increased C_8_^+^ hydrocarbon selectivity, achieving an optimized selectivity of 64.2%. With a sodium amount of 0.81 wt%, the C_8_^+^ selectivity of the modified CoFe catalyst was 2.4 times higher than that of the original catalyst, while the selectivity of CH_4_ was 4 times lower, indicating that the addition of Na has stronger effects on the suppression of CH_4_ production than the enhancement of C_8_^+^ production.

**FIGURE 5:**
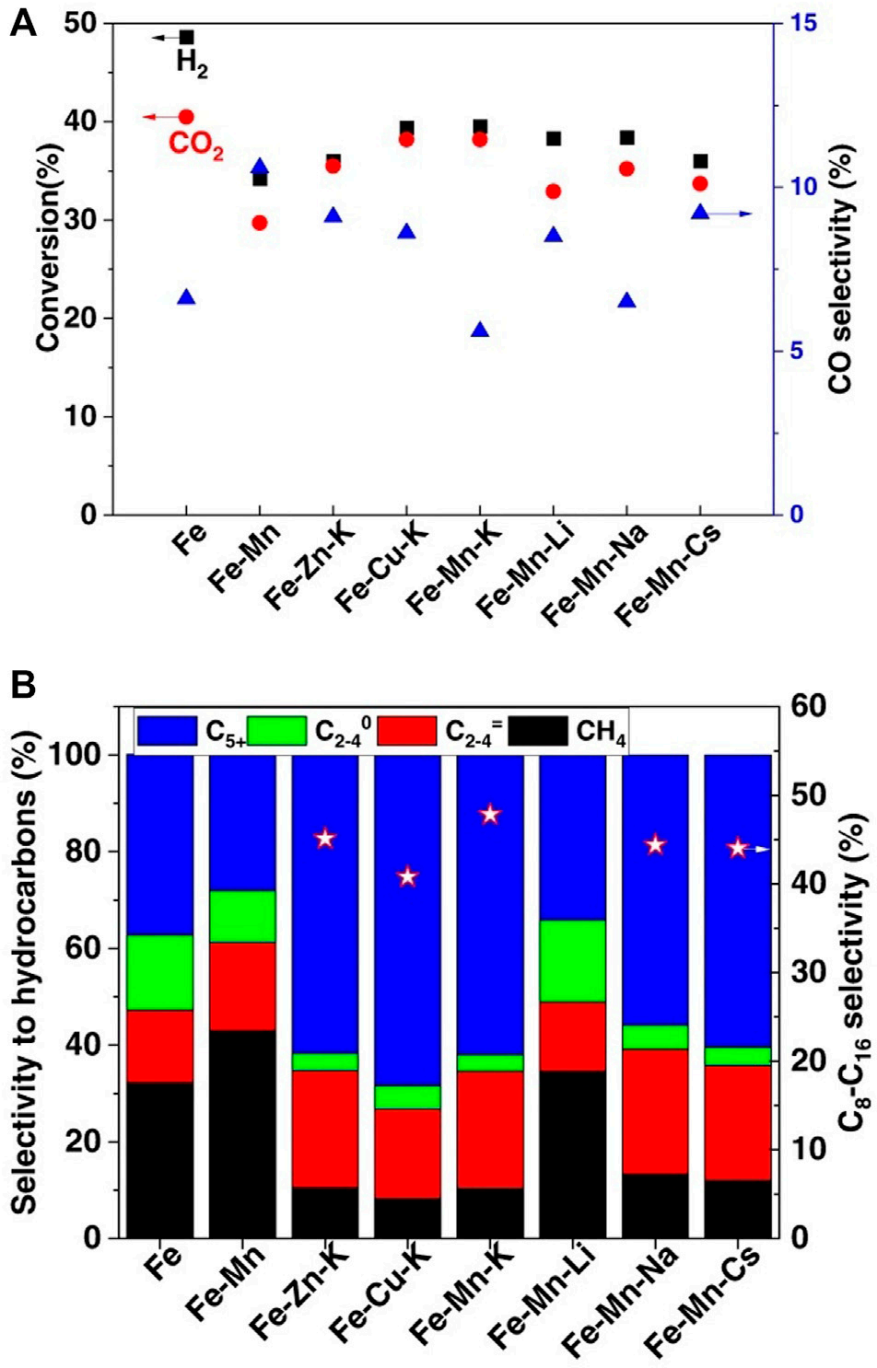
Catalyst performance for the hydrogenation of CO_2_ into jet fuel range hydrocarbons over various Fe-based catalysts (Yao et al., 2020) Copyright © 2020, The Authors. **(A)** CO_2_ conversion and CO selectivity under a reaction time of 20 h. **(B)** Selectivity for different hydrocarbon products under a reaction time of 20 h.

In addition to alkali metals, the addition of secondary metal could also enhance the production of long-chain hydrocarbons. For instance, cuprum and zinc are commonly used promoters as well, favorable for the reduction of Fe_2_O_3_ and the adsorption of CO_2_, which could increase the selectivity for C_5_^+^ hydrocarbons and decrease CH_4_ selectivity effectively (Satthawong et al., 2013; Zhai et al., 2016; Choi et al., 2017b; Liu et al., 2018b). Choi et al. (2017a) reported Fe-Cu catalysts that are used in the CO_2_ hydrogenation process. Under the optimized conditions of 1.0 MPa and 1800 ml/g·h, C_5_^+^ hydrocarbon reached the highest selectivity of 66.3%, while C_2_-C_4_ hydrocarbons and methane present a selectivity of 31% and only 2.7%, respectively. Yao et al. (2020) used the organic combustion method to prepare a series of Fe-based catalysts for the conversion of CO_2_ into jet fuel range hydrocarbons. Compared with Fe catalysts without promoters, Fe-Zn-K, Fe-Cu-K, and Fe-Mn-K catalysts presented better selectivity for the synthesis of jet fuels with lower methane and C_2_-C_4_ hydrocarbon selectivity. As for the effects of various base-metal promoters, all catalysts promoted by alkali metals presented great CO_2_ hydrogenation activity and jet fuel range hydrocarbons selectivity, despite the Li-modified catalyst which shows high methane selectivity but low selectivity for long-chain hydrocarbons. With a CO_2_ conversion of 38.2%, the optimized Fe-Mn-K catalyst showed a selectivity for C_8_-C_16_ hydrocarbons of 47.8% with a low selectivity for both CH_4_ and CO (Figure 5).

### 5.2 Catalyst supports

With unique steric properties including multidimensional structure, complex porosity, and appropriate acidity, zeolites have been widely utilized in isomerization reactions (Akhmedov and Al-Khowaiter, 2007; Lesthaeghe et al., 2007; Shamzhy et al., 2019; Miao et al., 2020), showing great potential for the synthesis of long-chain hydrocarbons from CO_2_ (Gao et al., 2017b; Ni et al., 2018; Wei et al., 2018; Zhang et al., 2019a; Wang et al., 2019). For instance, Soualah et al. (2008) studied the effects of structural properties of various zeolites (HZSM-5, HMCM-22, and HBeta) on the hydro-isomerization of long-chain alkane over a Pt catalyst. The results indicated that the diffusion of different reactants is significantly affected by the inside porosity and structural properties of zeolites in the catalyst, leading to an increment in the number of branches in each isomer. Wei et al. (2018) designed a novel Na-modified Fe_3_O_4_ catalyst coupled with various acidic zeolites, which presented greatly improved selectivity for liquid hydrocarbons, indicating that steric property is the most important factor influencing the selectivity for C_5_^+^ hydrocarbons for the catalysts coated with zeolites.

Based on the investigation results of the direct hydrogenation of CO_2_ to multibranched isomers (Liu et al., 2009; Zhang et al., 2019b; Cheng et al., 2020), Noreen et al. (2020) prepared a novel NaFe catalyst (Na/Fe_3_O_4_) coated on various acid zeolites including SAPO-11 and ZSM-5 for CO_2_ conversion into liquid hydrocarbons. With aid from sodium ion which is favorable for the formation of Fe_5_C_2_ and acid sites in catalyst, the NaFe catalyst presented a CO_2_ conversion of 32.9% and a C_5_^+^ hydrocarbon selectivity of more than 60%, which greatly enhanced *via* adding zeolites to the catalyst stream. Compared with the original catalyst, both the CO_2_ conversion and liquid hydrocarbon selectivity significantly increased due to the oligomerization reaction on the acid sites of zeolites, resulting in a decrease of light hydrocarbon content, achieving a maximum C_5_^+^ hydrocarbon selectivity of 75.5% (ZSM-5) and 72.3% (SAPO-11). However, the yield of C_5_^+^ hydrocarbons was reduced by combining different zeolites in the catalyst, showing that the activity of the isomerization reaction is affected by both the kind and mass of zeolites. As for selectivity for CO, no well-marked relationship could be found by research results, showing that RWGS activity is affected by NaFe instead of zeolite type.

### 5.3 Preparation and operation conditions

Similar to the catalysts used for producing other products, the selectivity for C_5_^+^ hydrocarbons in the CO_2_ hydrogenation process is influenced by the preparation methods of the catalyst. For instance, the catalytic performance of the catalyst system reported by Tan et al. was remarkably affected by the conditions of hydrothermal pretreatment, including hydrothermal time and temperature. With an increment of hydrothermal time, both the conversion of CO_2_ and selectivity for Oxy increased significantly, which greatly affects the distribution of various hydrocarbon products. On the other hand, the conversion of CO_2_ decreases slightly with the increment of hydrothermal temperature. Under an optimized temperature of 180°C and a weight ratio of TPABr to Fe-Zn-Zr of 1.0, the selectivity for isoalkane in C_5_^+^ hydrocarbon products reaches a maximum of 93%, which indicated the great importance of matching the content of metal oxides and ZSM-5 zeolite during the catalyst preparation process.

In addition to the preparation methods of catalyst, the distribution of various hydrocarbon products is greatly influenced by the operation conditions (reaction temperature, pressure, and time) as well. For instance, as an endothermal reaction, high temperature is beneficial to the RWGS reaction; it is favorable for the conversion of CO_2_ and the formation of CO. However, the selectivity of hydrocarbons decreased gradually with the increment of reaction temperature. Additionally, the isomerization reaction and hydrogen transfer of higher olefins also present great activity at high temperatures, resulting in a stable selectivity for C_5_^+^ isoalkanes and a decreased yield of aromatics at high reaction temperatures (340–370°C) (Figure 5D).

**FIGURE 6:**
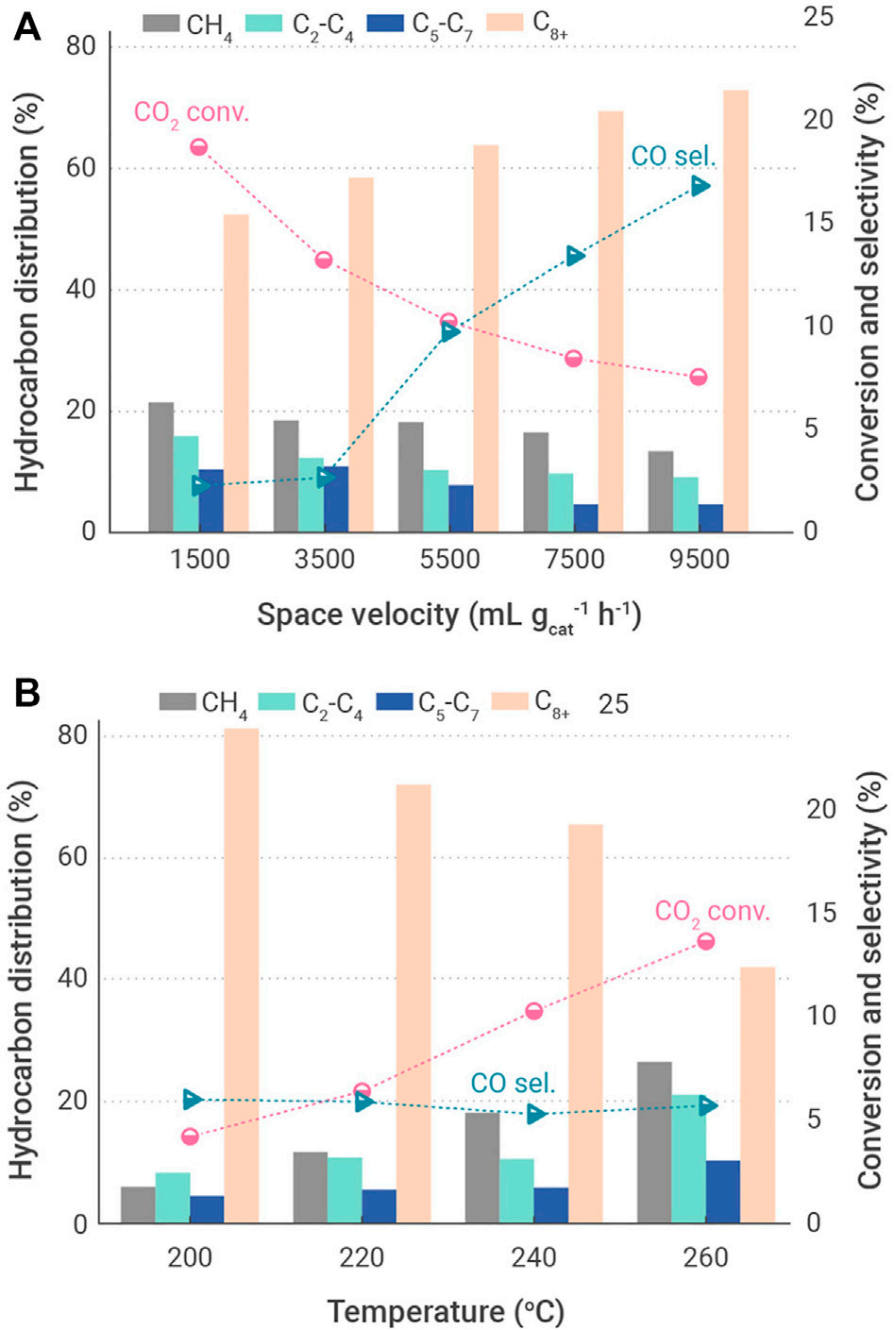
Effects of operation conditions on catalyst performance for the hydrogenation of CO_2_ into jet fuel range hydrocarbons over CoFe-0.81Na catalyst (Zhang et al., 2021). **(A)** Different reaction temperatures. **(B)** Different space velocities.

A similar result is obtained from the CO_2_ hydrogenation to jet fuel synthesis over NaFe catalyst, in which the activity was reduced with the decrease of reaction temperature (Figure 6). In order to suppress the activity of RWGS reaction, an optimal reaction temperature was found to be at 240°C, with a CO_2_ conversion of 10.2% and a selectivity for CH_4_ and C_8_-C_16_ hydrocarbons of 19% and 63.5%, respectively. As for the effects of space velocity on the catalytic performance, the selectivity for jet fuel range hydrocarbons increased remarkably with an increment of feed gas space velocity, reaching a maximum of 73.1%, while both the selectivity for CH_4_ and conversion of CO_2_ decreased significantly.

## 6 CO_2_ hydrogenation to aromatics

Amongst various products of CO_2_ hydrogenation, aromatics are one of the most valuable chemicals, which could be utilized as the raw materials of polymers, medicines, and petrochemicals (Niziolek et al., 2016). Currently, most aromatics come from the process of petroleum refining, including oil thermal cracking and naphtha reforming. Moreover, methanol-to-aromatics (MTA) is another crucial process of aromatics production (Zhang et al., 2014). With an increment rate of 6% per year, the increasing demand for high-valued materials synthesized from aromatics has attracted attention from both industry and scientific research, which, however, mainly depends on the energy-intensive petroleum and natural gas industry (Olsbye et al., 2012). Thus, it is imperative to propose an alternative process for preparing aromatics from CO_2_, providing an important method of developing valuable chemical production processes with relatively low cost. Recently, a variety of research studies have investigated CO_2_ hydrogenation for synthesizing aromatics. In this section, a comprehensive review of the aromatics production from CO_2_ from both RWGS-FTS and MeOH routes is provided, the emphasis of which is focused on the various modification approaches of different catalysts. Moreover, synthesis methods and optimization of reaction conditions are discussed as well. Some results of previous studies are shown in Table 2.

**TABLE 2:** Catalytic performances for the CO_2_ conversion into light aromatics.

Catalyst	H_2_/CO_2_ (molar)	Reaction temperature (°C)	Reaction pressure (MPa)	GHSV (gas hourly space velocity)	CO_2_ conversion (%)	Product selectivity (%)			Ref
						CO	C_2_^=^-C_4_^=^	Aromatics	
Fe_2_O_3_/KO_2_/ZSM-5	3.0	375	3.0	5,000 ml/g·h	48.9	N/A	12.1	24.9	Ramirez et al. (2019)
Fe_2_O_3_/KO_2_/MOR	3.0	375	3.0	5,000 ml/g·h	48.3	N/A	33.3	2.6	
ZnO/ZrO_2_/H-ZSM-5 (powder)	3.0	340	4.0	7,200 ml/g·h	∼16	∼35	∼6	∼74	Zhou et al. (2019b)
ZnO/ZrO_2_/H-ZSM-5 (60–100 mesh)	3.0	340	4.0	7,200 ml/g·h	∼11	∼30	∼10	∼64	
ZnO/ZrO_2_/H-ZSM-5 (dual bed)	3.0	340	4.0	7,200 ml/g·h	∼9	∼10	∼25	∼25	
ZnCr/H-ZSM-5 (SiO_2_/Al_2_O_3_ = 50)	3.0	350	5.0	2,000 ml/g·h	30.2	60.1	10.7	9.17	Zhang et al. (2019a)
ZnCr/ZnZSM-5 (SiO_2_/Al_2_O_3_ = 50)	3.0	350	5.0	2,000 ml/g·h	33.2	59.3	28.4	26.5	
ZnCr/H-ZSM-5 (SiO_2_/Al_2_O_3_ = 140)	3.0	350	5.0	2,000 ml/g·h	31.6	71.0	N/A	65.1	
ZnCr/Zn/ZSM-5 (SiO_2_/Al_2_O_3_ = 140)	3.0	350	5.0	2,000 ml/g·h	30.5	60.8	N/A	62.3	
ZnAlO_x_/H-ZSM-5 (mixing)	3.0	320	3.0	6,000 ml/g·h	∼5.1	∼45	∼14	∼55	Ni et al. (2018)
ZnAlO_x_/H-ZSM-5 (dual bed)	3.0	320	3.0	6,000 ml/g·h	∼4.7	∼57	∼9	∼20	
ZnAlO_x_/H-ZSM-5 (grinding)	3.0	320	3.0	6,000 ml/g·h	∼5.4	57.4	∼15	∼65	

**FIGURE 7:**
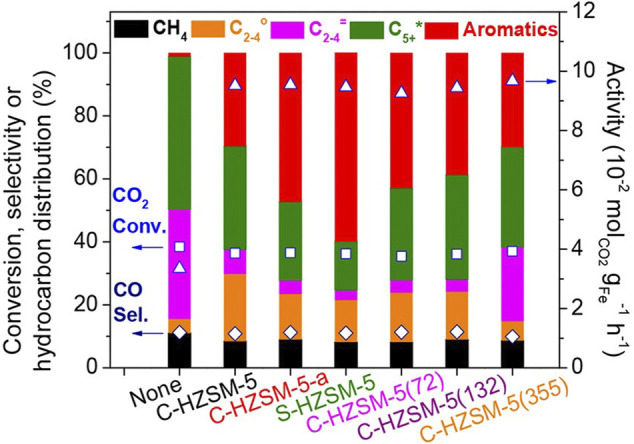
CO_2_ conversion and product selectivity over sole ZnFeO_x_-4.25Na or composite catalysts containing ZnFeO_x_-4.25Na and various zeolites (Cui et al., 2019).

Compared with the traditional FTS catalysts which produced products presenting broader carbon number distributions but relatively low aromatics selectivity, catalysts loaded on ZSM-5 presented higher aromatics selectivity, showing great ability to facilitate the aromatization of olefins (Tackett et al., 2019). For instance, the n- and iso-hydrocarbons produced on active sites in Fe catalyst could be diffused into inner channels of HZSM-5, taking part in the sequential aromatization reactions, which results in the high aromatics yield and relatively low paraffin selectivity of Fe catalyst loaded on HZSM-5 zeolite (Cui et al., 2019). In addition to Fe, composite catalysts containing other metals or metal oxides (such as Zn and Na) and different kinds of zeolites also showed great CO_2_ conversion and selective hydrocarbon yield (Figure 7).

Combined with simulation results of density functional theory calculations, the activity of aromatization reaction is greatly affected by the Brønsted acidity of catalysts, which is the key factor of the breaking of C-C bond in n-heptane and subsequent aromatization reactions. Consequently, the HZSM-5 with a great number of acid sites is favorable for the formation of active phases in catalysts (such as Fe_3_O_4_ and χ-Fe_5_C_2_), which favors the formation of aromatics from CO_2_ hydrogenation. In addition, the external surface acidity of HZSM-5 also affects the isomerization of aromatic products. For instance, a composite catalyst of SiO_2_ and HZSM-5 eliminated external Brønsted acid sites *via* the hydrothermal heating method, enhancing the selectivity for p-xylene from 24% to 70% (Xu et al., 2019).

In addition to the amounts of Brønsted acid sites, the aromatization reaction of hydrocarbons is also influenced by the ratio of SiO_2_/Al_2_O_3_ in ZSM-5. The study investigated by Ramirez and co-workers reported that the selectivity of total aromatics, associated with the acidity of the catalyst, decreased with the increment of Si/Al ratio in the catalyst (Ramirez et al., 2019). Furthermore, the research results indicated that the selectivity for aromatics was increased by using ZSM-5, whereas mordenite (MOR) was unfavorable for the production of ethylene and propylene. By comparison, ZSM-5 with the Mobil Five (MFI) presents a much better ability to convert olefins to aromatics by improving the generation activity of carbenium ions, the key intermediates for aromatization, from olefins. Furthermore, MOR contains more protonation barriers than ZSM-5, resulting in the worse activity in the aromatization of heavy olefin fraction.

As for the CO_2_ hydrogenation *via* the MeOH route, the modification of zeolite structures is an important way to increase the molar ratio of olefins to aromatics in products by facilitating the diffusion of reaction intermediates toward zeolites. For instance, the reduction of crystal size is favorable for the promotion of mass transfer in catalysts, leading to the gradual increment of olefin/paraffin (O/P) ratio and olefin selectivity. Thus, some researchers utilized TEA and TEAOH templates pretreated with nitric acid to prepare SAPO-34 zeolites with micropores, mesopores, and macropores, which presented high porosity and greatly improved mass transfer and olefin selectivity (Nishiyama et al., 2009).

As the key factor that affects the catalytic performance of secondary hydrogenation reactions, the amount and concentration of Brønsted acid sites on zeolites are considerable parameters of a CO_2_ hydrogenation catalyst to control the hydrocarbon distribution in the products (Kanai et al., 1992; Chen et al., 2008). In addition to the nitric acid treatment of SAPO-34 (Song et al., 2017), using zeolite loaded with Zn is another important method to increase olefin selectivity by reducing the acidity of zeolite (Chen et al., 2019b). For instance, the CO_2_ hydrogenation process using ZSM-5 doped with Zn *via* ion exchange showed greatly improved aromatics selectivity due to excessive hydrogenation suppressed by the reduced acidity, leading to the generation of long-chain hydrocarbons (Zhang et al., 2019a). Additionally, the increment of SiO_2_/Al_2_O_3_ ratio in ZSM-5 is favorable for the reduction in surface acid density and formation of C_5_^+^ hydrocarbons (including aromatics). For example, with the increment of SiO_2_/Al_2_O_3_ ratio, the selectivity of olefins and aromatics was increased in the CO_2_ hydrogenation process using Co/H-ZSM-5 catalysts promoted by K. In addition to the composite of zeolite, metal oxide in the bifunctional catalyst is another key factor that greatly affects the Brønsted acid sites of catalyst (Kanai et al., 1992; Senger and Radom, 2000; Cheng et al., 2016). For instance, based on the results of DTBPy-FTIR characterizations, Ni et al. reported that ZnAlO_x_ suppressed the Brønsted acid sites on the surface of H-ZSM-5 zeolite after a series of treatments, including mixing, grinding, and high-pressure pressing (Ni et al., 2018). Furthermore, the ZSM-5 with core-shell (H-ZSM-5@S-1), which could decrease the number of acid sites on the surface of H-ZSM-5, showed greatly improved selectivity for benzene, toluene, and xylene (BTX) (Wang et al., 2018c).

As for the deactivation of zeolite catalysts, coke formation on the surface of the catalyst is a typical serious deactivation process (Zhang et al., 2019c). For instance, with a working time of less than 20 h, a lot of coke was generated on the surface of the CuO-ZnO-Al_2_O_3_/SAPO-34 catalyst, which blocked pores and covered acid sites of zeolites, resulting in the decrease of activity (Tian et al., 2020). However, accurate kinetics of the coking process on the catalyst with small pores is difficult to establish. In order to prevent relevant effects, a series of methods have been employed to inhibit the formation of coke, including using zeolites with cuboid morphology and reducing the crystal size of zeolites (Dang et al., 2019). For instance, the coke on the surface of SAPO-34 zeolite from the spent bifunctional catalyst is full of precursor species (Sun et al., 2015; Wang et al., 2015), which escaped from the zeolite cages with hierarchical structures and subsequently inhibited the formation of polycyclic aromatics, prolonging the life span of catalysts. In other research studies, zeolite with smaller crystal sizes, which is favorable for diffusing the coke precursor, is used for catalyst preparation to slow the coke rate (Dang et al., 2019).

In order to improve the selectivity and yields of olefin or aromatics, it is imperative to enhance the proximity of various active components, which could be achieved by various methods (Wang et al., 2018c; Ni et al., 2018; Ramirez et al., 2019). For instance, the ZnO/ZrO_2_ catalyst loaded on H-ZSM-5 using a dual bed presented the lowest conversion of CO_2_ and selectivity of aromatics, which could be improved by shortening the spatial distance of different active components. By using motor mixing to shorten the distance between two catalytic components in the catalyst system, the CO_2_ reached the highest conversion of 16% with an aromatics selectivity of 76%, indicating that a shorter distance between active components is favorable for aromatics generation (Zhou et al., 2019b). Additionally, the distance between the two active components could also be shortened *via* using zeolite with a core shell. Compared with the traditional CuZnZr/Zn/SAPO-34 system prepared *via* physically mixing, which is favorable for the production of CH_4_ and detrimental to the generation of low olefins, the novel system with core-shell structure (CuZnZr@Zn-SAPO-34) produced olefins as the main product instead of CH_4_. With the aid of core-shell-structured zeolite, the proximity between the metal oxide and zeolite was reduced significantly, which is unfavorable for the further hydrogenation of hydrocarbons, resulting in the increase of olefin and aromatics selectivity (Weisz, 1962; Chen et al., 2019b).

Suppressing the RWGS reaction is an efficient method to increase the olefin/aromatics selectivity in the MeOH route due to the large amount of CO generated in the RWGS reaction during the CO_2_ hydrogenation process (Gao et al., 2017a; Chen et al., 2019b; Ma and Porosoff, 2019). By controlling appropriate reaction conditions, the CO formation during the CO_2_ hydrogenation process could be suppressed over the In_2_O_3_-ZrO_2_/SAPO-34 catalyst (Tan et al., 2019). For instance, the appropriate reaction temperature of the CO_2_ hydrogenation process is optimized as 573 K to decrease CO selectivity from more than 80% to only 13%. Additionally, the CO selectivity achieves 5% by increasing the space velocity of the H_2_/CO_2_ ratio in the feed gas. By increasing the H_2_ content from 3/1 to 9/1, the reaction rate of CO_2_ hydrogenation increases by around 60%, while the selectivity of aromatics in products decreases significantly. Furthermore, the addition of a small amount of CO into the feed gas could further inhibit CO formation, resulting in a CO selectivity of only 2% (Ni et al., 2018).

In consideration of the high cost of H_2_ production, it is significant to decrease the H_2_ consumption in the CO_2_ hydrogenation process. However, few researchers pay attention to the activation of H_2_, compared to the improvement of ability to adsorb and activate CO_2_ (Chen et al., 2019c). Chen et al. used carbon-limited MgH_2_ nanolayer catalysts in the CO_2_ hydrogenation process to reduce H_2_ consumption (Chen et al., 2019d). At the beginning of the process, lattice H^−^ was formed in the lattice of MgH_2_ to prevent the formation of aromatic H, which then combined with C from CO_2_ molecule to produce formate intermediate, which was then subsequently hydrogenated to form olefins at lower H_2_/CO_2_, achieving the decrease of H_2_ consumption. Compared with a common CO_2_ hydrogenation reaction system with an H_2_/CO_2_ ratio of 3, the MgH_2_ catalyst system could prepare olefins and aromatics with high selectivity (about 50%) at low H_2_ partial pressure (H_2_/CO_2_ = 1:8). However, the MgH_2_ catalyst system presents low CO_2_ conversion (only 10%).

## 7 Conclusion and future opportunities

In recent years, CO_2_ content in the atmosphere is increasing at an unprecedented rate, which has been a global concern due to associated foreseen and colossal damages of global warming, leading to an increasingly loud voice accelerating the reduction of excessive CO_2_ emission to achieve carbon emission peak and carbon neutrality as soon as possible. Thus, a great variety of research efforts have been carried out on the process of CO_2_ hydrogenation all over the world to curb this challenge, aiming at utilizing CO_2_ by efficient conversion into high-valued hydrocarbon products such as olefins, fuels, and aromatics. By developing a novel process of CO_2_ utilization and providing a novel pathway for sustainable production of high-value chemicals, the ever-growing atmospheric CO_2_ content and the global warming caused by excessive CO_2_ emissions could be solved as well.

Amongst various CO_2_ hydrogenation processes, MeOH and RWGS-FTS routes are the two main methods for converting CO_2_ into hydrocarbon products. With advantages such as being more economical and environmentally acceptable, considerable research progress has been made on one step to convert CO_2_ into hydrocarbons, the so-called “direct hydrogenation process”. During the hydrogenation process, conversion of CO_2_ and selectivity of target hydrocarbon product are influenced by various factors such as the composition and stability of catalysts. However, there are still many challenges in the development of the CO_2_ hydrogenation process, requiring enormous further efforts from the academic and industrial world. The important points for future consideration for the improvement of the activity and selectivity are shown below.

### 7.1 Modification of composition and structures of catalysts

Compared with the traditional CO_2_ hydrogenation process using molecular H_2_, lattice H^−^ generated by metal hydrides provides a novel method for hydrogenation reactions, showing great potential for further investigations, the main challenge of which lies in the preparation of stable metal hydrides. Amongst different metal hydrides, gold and copper hydrides have shown great potential to be used in the catalytic system for CO_2_ conversion into olefins and other hydrocarbons due to their great stability, synthetic versatility, and abundant reserves (Jordan et al., 2016). In addition to hydride, metal carbide is another composition that greatly affects the CO_2_ hydrogenation process. For instance, iron carbide, which is prepared from carbon materials supported by Fe-based catalysts, is an active phase for CO intermediate hydrogenation in the FTS process. Thus, the performance of hydrogenation catalysts could be further improved by exploring novel methods for producing stable and active metal hydrides and carbides.

As one of the currently most used catalyst materials in the CO_2_ hydrogenation process, exploring efficient methods of tuning the shape selectivity of zeolite to control the distribution of hydrocarbon products is an essential direction for current research. For instance, developing zeolites with specific structures or improving the mass transfer of chemicals in the system has been a hot topic in recent studies. In addition, developing zeolite preparation methods by using novel materials is another hot investigation topic due to the large amounts of reagents consumed in the molecular sieve preparation, including aluminum isopropoxide, silica sol, and other purification chemicals, resulting in the high cost of zeolite. In recent studies, novel synthesis methods of SAPO-34 and ZSM-5 zeolites have already been reported by using different raw materials, including palygorskite, kaolin, diatomaceous earth, and Thai perlite (Tangkawanit et al., 2005; Wajima et al., 2008; Wang et al., 2018d). For instance, CuO-ZnO-Al_2_O_3_ mixed oxides loaded on a novel SAPO-34, which presents a larger surface area by adding palygorskite, show a better activity of CO_2_ hydrogenation compared with conventional synthesis methods (Tian et al., 2020).

In order to obtain selective products, it is important to control the extent of reactions in each stage. In consideration of the effects of Brønsted acid sites on secondary hydrogenation, passivating excess Brønsted acid sites of zeolite under the premise of an active catalyst provides a useful method for improving selectivity and yield for selective products, especially for olefins or aromatics. For instance, a series of modification methods have been employed for zeolite preparation such as the addition of Zn (Zhang et al., 2019a) and the increase of SiO_2_/Al_2_O_3_ ratio in raw materials (Chen et al., 2019b), using alkali metals, alkaline oxides, and nitric-acid pretreatment (Dang et al., 2019). Furthermore, preparing zeolites with reagents such as tetrachloride and ammonium fluorosilicate or under calcination temperatures could adjust the acidity on the surface of zeolite as well.

As a popular catalyst that has been widely used in the CO_2_ hydrogenation process, the interaction of the two active components in bi-functional catalysts plays an important role in suppressing the formation of CO, which is beneficial to the production of selective hydrocarbons, both *via* RWGS-FTS and MeOH routes. The main challenge in such bi-functional systems is the high selectivity of CO and CH_4_ instead of desirable hydrocarbon products. Generally, smaller particles with more uniform mixing had positive effects on CO_2_ conversion and selectivity for aromatic hydrocarbons, which leads to a closer distance between different components (Zhou et al., 2019b). Recent investigations report that zeolite with a core-shell structure prepared by the carbon sacrificial method had a thinner metal shell, which was beneficial to the dispersion of active sites, leading to the enhancement of the interaction between various active sites (Li et al., 2020). Thus, more attention should be paid to improving the interaction between different components and novel effective mixing methods.

An ineluctable problem faced by the CO_2_ hydrogenation process lies in the deactivation of catalysts resulting from the formation of coke on the surface of the catalyst. For instance, the extended polyenes are likely to block the zeolite pores, making them much easier to deactivate, which, however, could be avoided by the MFI structure of zeolites, inhibiting the cyclization of polyenes. Additionally, hydroxyl groups formed by water on the surface of zeolites also deactivate the catalysts (Fröhlich et al., 1996; Porosoff et al., 2016). Thus, recent studies have focused on the regulation of intrinsic basicity and hydrophobicity of zeolites, aiming at balancing the hydrophobic and hydrophilic properties. For example, the increment of the SiO_2_/Al_2_O_3_ ratio in zeolites is an efficient method to improve the hydrophobic properties. Hydrothermal treatment is another effective method to improve hydrophobicity *via* dealumination, as reported by recent research (Lee et al., 2020). In short, developing a preparation method to synthesize catalysts tolerant to water and coke formation is a promising research direction in the future.

### 7.2 Simulation and calculations

As an efficient method for investigating the mechanisms of complicated reactions, DFT calculations could model the molecular behavior of various reagents during the CO_2_ hydrogenation process. However, the model of various components was established based on bulk properties and average parameters, which could not reflect the real characteristics of the reaction, requiring the researcher to establish relevant structural models by combining DFT calculation results with characterization experiments (Ma and Porosoff, 2019). In addition to accurate characterization, micro-kinetic simulations and kinetic Monte Carlo (KMC) are also widely used tools to correct the models of DFT calculations, correlating theoretical results with experimental results of catalytic hydrogenation from CO_2_ to selective hydrocarbons (Kattel et al., 2017). Furthermore, machine learning has attracted great attention from CO_2_ utilization due to its higher speed and lower cost than traditional computational methods, aiming at understanding and predicting complex reaction mechanisms (Goldsmith et al., 2018; Kitchin, 2018; Gusarov et al., 2020; Smith et al., 2020). To sum up, all the theoretical approaches mentioned earlier show great potential to be applied in the CO_2_ conversion process to hydrocarbon products.

### 7.3 Inhibition of deactivation and improvement of stability

As a great challenge for the CO_2_ hydrogenation process, the catalysts are likely to be deactivated *via* several ways like sintering, phase transformations (Li et al., 2018), and poisoning by the formation of coke and water (Rytter and Holmen, 2017; Arora et al., 2018a), leading to the reduction of CO_2_ conversion and desirable product selectivity. Thus, it is imperative to inhibit the deactivation of catalysts, requiring various methods for different forms of deactivation. For instance, it is efficient to prevent sintering and agglomeration by anchoring nanoparticles on Al sites (Gao et al., 2014) and using promoters to stabilize them.

In addition, the coke generated by hydrocarbon products is another unavoidable challenge for the CO_2_ hydrogenation process, the extent of which could be determined by temperature-programmed oxidation (TPO) and Raman spectroscopy (Chua and Stair, 2003). In order to mitigate the deactivation of catalysts caused by coke, especially for the zeolite-based catalysts with a rapid deactivation rate, various methods could be applied such as reducing the diffusion limitations of catalysts by shortening diffusion paths or extending the life span of zeolites (Yang et al., 2014).

Water formation and carbon deposition are also important factors influencing the stability of catalysts, the deactivation of which is more difficult to prevent. As the main byproduct of the CO_2_ hydrogenation process, water is generated from the oxygen atoms derived from the dissociation of CO_2_, causing great damage to the catalytic performance by causing crystallinity loss or modifying acid sites (Barbera et al., 2011; Zhang et al., 2015b), which could be inhibited by varying the acidic properties of zeolites (Deimund et al., 2015; Liang et al., 2016). Like coke deposition, the deactivation of water could also be mitigated by synthesizing nano or hierarchical zeolites (Yang et al., 2014). For example, ZSM-5 zeolite with mesopores could be prepared with the aid of polymeric templates such as polyethylene glycol (PEG) (Tao et al., 2013), which presents greatly improved mass transfer.

In addition, recent studies using improved equipment or well-designed processes to remove excess water have shown a good promotion effect on CO_2_ hydrogenation (Anicic et al., 2014; Cui and Kær, 2019). For instance, Joo et al. (1999) initially reported a two-step process converting CO_2_ into methanol (carbon dioxide hydrogenation to form methanol *via* a reverse water–gas shift reaction, CAMERE process), in which the RWGS reaction was added to consume water byproduct, achieving an increase in the yield of methanol. In consideration of the high operating temperature required by the endothermic RWGS reaction, the *in situ* water removal (ISWR) is utilized to shift the thermodynamic equilibrium, lowering the operating temperature and enabling the CAMERE process to work at moderate temperatures (Zachopoulos and Heracleous, 2017), which could be achieved by using membrane reactors or developing a novel reaction process to enhance water sorption.

The membrane reactors with ISWR have been used for various processes (Gallucci et al., 2004; Gallucci and Basile, 2007; Iliuta et al., 2010; Diban et al., 2013; Atsonios et al., 2016; Zhou et al., 2016; De Falco et al., 2017; Catarina Faria et al., 2018; Gorbe et al., 2018) including the CO_2_ hydrogenation process, showing great potential for the improvement of CO_2_ utilization. With the aid of a zeolite A membrane, Gorbe et al. (2018) studied the hydrogenation process from CO_2_ to methanol. Under the operation conditions of 100–270 kPa and 160–260°C, the membrane reactor presented a brilliant ability to separate water byproducts from complex gas mixtures, showing the potential of a membrane reactor used in a moderate temperature RWGS process. The other utilization method of ISWR is the sorption-enhanced reaction process (SERP), in which sorbent is used to remove byproduct water (Iliuta et al., 2011; Dehghani et al., 2014; Arora et al., 2018b). For instance, the conversion of CO_2_ to CO increased from less than 10% to a maximum of 36% at a low reaction temperature by using a bench-scale SERP (Carvill et al., 1996). Under a reaction temperature of 225–250°C and a pressure of 5–10 bar, the CO_2_-to-CO conversion achieved a three-time increment after the application of SERP (Jung et al., 2013).

It should be noted that the utilization of membrane reactors and SERP in the CO_2_ hydrogenation process is still at the research stage, which is mainly limited by cost issues. Up to now, relevant studies still lack cost evaluations for membrane reactors. Additionally, the membrane reactor is more complex than a traditional reactor, resulting in a higher equipment cost (Atsonios et al., 2016). To sum up, the deactivation of the CO_2_ hydrogenation process could be mitigated by using the strategies shown earlier.

### 7.4 Optimization of operating conditions

As an important factor in the CO_2_ conversion process, reaction conditions could affect the activity of CO_2_ hydrogenation and the selectivity for various hydrocarbon products by controlling the reaction extent. For instance, a higher reaction temperature is favorable for CO_2_ hydrogenation and an increase of CH_4_ selectivity, requiring to find an optimal temperature to balance CO_2_ conversion and the yields of selective hydrocarbons, except for methane. Additionally, CO_2_ conversion increases with the increment of reaction pressure, which is favorable for the production of hydrocarbons. However, secondary hydrogenation occurs at high pressure, especially under the high partial pressure of H_2_, leading to a decrease in the olefin/paraffin ratio, which is unfavorable for the production of olefins. Thus, choosing an appropriate reaction pressure is crucial for the hydrogenation process, combining the improvement of conversion and selectivity and reduction of capital costs. Meanwhile, process development requires mild conditions to lower the energy cost of reaction, which needs researchers to develop CO_2_ hydrogenation catalysts with great activity under moderate or ambient pressures, which can be achieved by adding alkali promoters (such as Na and K) (Zhang et al., 2015a; Gao et al., 2017a) and specific oxide supports (Torrente-Murciano et al., 2016; da Silva and Mota, 2019) and using molecular sieves that allow low-pressure reactions. In conclusion, more studies are needed to optimize the operation conditions to improve the yield toward desirable products.
